# Enhanced Detection of Viral RNA Species Using FokI-Assisted Digestion of DNA Duplexes and DNA/RNA Hybrids

**DOI:** 10.1021/acs.analchem.2c00407

**Published:** 2022-04-25

**Authors:** Juan R. Tejedor, Gabriel Martín, Annalisa Roberti, Cristina Mangas, Pablo Santamarina-Ojeda, Raúl Fernández Pérez, Virginia López, Rocío González Urdinguio, Juan J. Alba-Linares, Alfonso Peñarroya, Marta E. Álvarez-Argüelles, José A. Boga, Agustín Fernández Fernández, Susana Rojo-Alba, Mario Fernández Fraga

**Affiliations:** † Nanomaterials and Nanotechnology Research Center (CINN-CSIC), El Entrego 33940, Spain; ‡ Foundation for Biomedical Research and Innovation in Asturias (FINBA), Oviedo 33011, Spain; § University Institute of Oncology (IUOPA), University of Oviedo, Oviedo 33006, Spain; ∥ Center for Biomedical Network Research on Rare Diseases (CIBERER), Madrid 28029, Spain; ⊥ Central University Hospital of Asturias (HUCA), Oviedo 33011, Spain; # Health Research Institute of Asturias (ISPA), Oviedo 33011, Spain

## Abstract

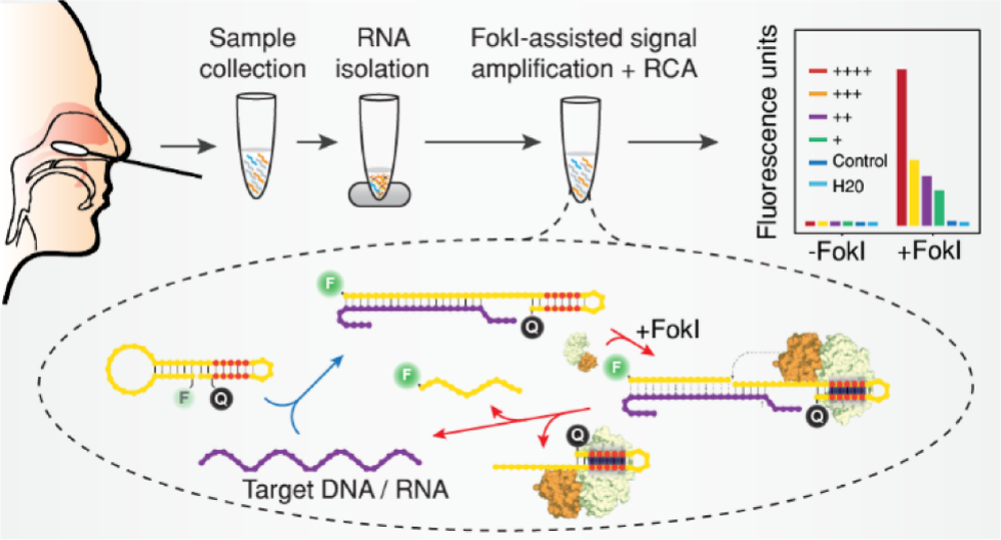

The accurate detection of nucleic acids from certain biological pathogens is critical for the diagnosis of human diseases. However, amplified detection of RNA molecules from a complex sample by direct detection of RNA/DNA hybrids remains a challenge. Here, we show that type IIS endonuclease FokI is able to digest DNA duplexes and DNA/RNA hybrids when assisted by a dumbbell-like fluorescent sensing oligonucleotide. As proof of concept, we designed a battery of sensing oligonucleotides against specific regions of the SARS-CoV-2 genome and interrogated the role of FokI relaxation as a potential nicking enzyme for fluorescence signal amplification. FokI-assisted digestion of SARS-CoV-2 probes increases the detection signal of ssDNA and RNA molecules and decreases the limit of detection more than 3.5-fold as compared to conventional molecular beacon approaches. This cleavage reaction is highly specific to its target molecules, and no detection of other highly related B-coronaviruses was observed in the presence of complex RNA mixtures. In addition, the FokI-assisted reaction has a high multiplexing potential, as the combined detection of different viral RNAs, including different SARS-CoV-2 variants, was achieved in the presence of multiple combinations of fluorophores and sensing oligonucleotides. When combined with isothermal rolling circle amplification technologies, FokI-assisted digestion reduced the detection time of SARS-CoV-2 in COVID-19-positive human samples with adequate sensitivity and specificity compared to conventional reverse transcription polymerase chain reaction approaches, highlighting the potential of FokI-assisted signal amplification as a valuable sensing mechanism for the detection of human pathogens.

Molecular approaches are powerful tools in pathogen discovery and aid in the diagnosis of human disease. The recent pandemic of COVID-19, caused by the novel human Beta coronavirus SARS-CoV-2,^1^ is an extreme example of how a viral pathogen can overwhelm the diagnostic capacity of most national healthcare systems. The current gold standard method for SARS-CoV-2 detection relies on the quantitative real time, reverse transcription polymerase chain reaction (RT-PCR) technique.^2^ While this technology is widely implemented and excels in terms of its specificity and sensitivity, it does require specialized equipment and has revealed certain drawbacks.^3^ Consequently, alternative approaches amenable to scalable purposes or point-of-care systems are required in order to facilitate the real-time surveillance of the disease.^4,5^

Adequate signal quantification is a critical parameter for accurate pathogen diagnosis. As such, most classical approaches have relied on the use of the versatile molecular beacon technology.^6^ In order to amplify the detection signal of nucleic acid targets, several techniques have combined the use of different enzymatic activities with the design of specific molecular beacons, giving rise to elegant isothermal amplification approaches. Some successful examples are nucleic acid sequence-based amplification,^7^ loop-mediated isothermal amplification (LAMP),^8^ rolling circle amplification (RCA),^9^ and nicking endonuclease signal amplification (NESA) methods,^10^ among others. While most of these approaches have proven to be useful in detecting target molecules, they all depend on the amplification of the nucleic acid content rather than on the amplification of the detection signal itself, and the enhanced detection is often hindered by the presence of high background levels caused by unwanted sources of amplification.^11^ On the other hand, signal amplification mediated by NESA does allow for the appropriate discrimination of DNA molecules in complex samples and dramatically reduces the limit of detection as compared to other conventional approaches.^12^ However, direct detection of RNA molecules, by means of enhanced detection of DNA/RNA heteroduplexes, is still challenging due to the lack of knowledge about enzymatic activities that can induce asymmetric cleavage of DNA/RNA hybrid strands.

The discovery of a set of restriction endonucleases (REs) able to hydrolyze DNA/RNA heteroduplexes paved the way for the development of novel approaches focused on the study of DNA/RNA hybrids.^13^ Previous evidence suggested that FokI, a type IIS RE, can perform the asymmetric digestion of DNA/RNA hybrids when the catalytic domain of the endonuclease is fused to specific zinc finger DNA-binding domains.^14^ FokI is an unusual restriction enzyme that recognizes a specific DNA sequence (GGATC) and cleaves non-specifically 9–13 bp away from the recognition site.^15^ Interestingly, previous work has highlighted the role of FokI as a primigenial genome editing tool when combined with custom adapter-primers.^16^ Second and third generation editing technologies, created from the combination of FokI cleavage domain with either zinc finger domains^17^ or the recent CRISPR/Cas9 technology,^18^ have also highlighted the potential of FokI as a powerful tool for genetic manipulation. In addition, a DNA/FokI-based replicating cutting machine was successfully used for the amplified signal detection of DNA analytes,^19^ thus demonstrating the high versatility of this RE for the detection of particular DNA targets.

These observations led us to hypothesize that FokI could be used as a sensing molecular tool to mediate the direct signal amplification of DNA/RNA hybrids, with potential implications for the enhanced detection of RNA-based pathogens. In this work, we demonstrated that the custom design of sensing dumbbell-like oligonucleotides can induce native FokI relaxation, resulting in the FokI-assisted digestion of DNA duplexes as well as DNA/RNA hybrids. Detection of synthetic RNA molecules corresponding to the Nsp4, Spike, and Orf8 genomic regions of SARS-CoV-2 was enhanced in the presence of the FokI-mediated sensing tool. The cleavage reaction was highly specific to its target molecules and can be multiplexed to facilitate the detection of other human B-coronaviruses including various SARS-CoV-2 variants. Simultaneous coupling of the FokI-assisted digestion technology with isothermal rolling circle amplification improved the limit of detection of the reaction and yielded an adequate specificity and sensitivity when compared to the conventional RT-qPCR technique. These results highlight the potential of the FokI-assisted digestion of DNA duplexes and DNA/RNA hybrids as a valuable signal amplification technology for the detection of RNA molecules with pathogenic potential for humans.

## Materials and Methods

### Strand-Specific Analysis of FokI/BanI Endonuclease Activity

Various combinations of synthetic RNA and DNA sequences including FokI and BanI restriction sites were designed and labeled with either IRD800 or Cy5.5 fluorophores at their 5′ end. All oligonucleotides used in this study (Table S1) were synthetized by Metabion (Germany). Annealing reactions included a DNA duplex or DNA/RNA heteroduplex mixture at a concentration of 1 μM in a final volume of 100 μl of RNAse-free water. Restriction enzyme digestions were performed at 37 °C for 1, 5, 10, 20, 30, 60, and 90 min in a reaction mix comprising 2 μl of NEB 10X Cutsmart buffer (NEB #B7204), 50 nM (1 pmol) of the duplex or heteroduplex substrate, and either 5 units of the RE FokI (NEB, #R0109S) or 10 units of BanI (NEB, #R0118S) in a final volume of 20 μl. Further details of this experimental protocol are provided in the Supporting Information.

### Conventional Hybridization Reactions and FokI-Assisted Signal Amplification Assays

All reactions were performed at 37 °C in a final reaction volume of 20 μL. For the conventional hybridization assays, the reaction mixtures contained 2 μL of NEB 10X Cutsmart buffer, 50 nM of custom dumbbell-like oligonucleotides for the different SARS-CoV-2 synthetic regions, and decreasing concentrations of either target DNA, target RNA (5 nM, 500, 50 pM, 0 M), or 50 nM of control DNA or RNA for each corresponding reaction (10-fold excess). A basic FokI-assisted signal amplification assay was performed as indicated in the conventional hybridization protocol but including the addition of 5 units of FokI RE. An extended FokI signal amplification assay was performed as indicated in the basic FokI-assisted signal amplification assay but was carried out in the presence of 50 nM universal hairpin oligonucleotide (UB). The fluoresce intensity of the reaction mixtures was recorded over time using a StepOnePlus Real-Time PCR System (Applied Biosystems). The excitation and emission wavelengths were configured to detect the FAM channel (488 and 520 nm, respectively). The reactions were maintained for 90 cycles (each cycle 1 min at 37 °C), and fluorescence measurements were obtained in each of the cycles.

### Padlock Probe Circularization and Molecular Coupling between FokI-Assisted Signal Amplification and RCA Technologies

Padlock probes containing a phosphate modification at their 5′ end were hybridized with their corresponding synthetic RNA molecules, and the padlock probe circularization step was performed in a reaction volume of 8 μL. The concentrations of the reaction components were 0.8 μL of NEB 10× SplintR ligase reaction buffer, 5 units of SplintR ligase (NEB, #M0375S), 10 nM of padlock probe oligonucleotides, and variable amounts of each target RNA molecule (1 fmol, 100, 10, 1, 0 amol), with the exception of the control RNA condition, which was always assayed at 1 fmol. This reaction mixture was incubated for 5 min at room temperature, and then, 12 μL of an RCA premix was added to the resulting reaction. The concentrations and amounts of the RCA premix components were 2 μL of NEB 10× Cutsmart buffer; 40 nM of the universal rolling circle primer; 500 μM dATP, dGTP, dCTP, and dTTP (Promega, #U1240); 0.5 μL of QualiPhi DNA polymerase (4Basebio, #510100); 0.01 units of pyrophospatase inorganic from *Escherichia coli* (NEB, #M0361S); 100 nM of 5′ FAM and 3′BHQ-1 labeled dumbbell-like oligonucleotides; and 5 units of FokI RE, yielding a final reaction volume of 20 μL. Fluorescence intensity in the FAM channel was recorded over 120 cycles (each cycle 1 min at 37 °C) in a StepOnePlus Real-Time PCR System, as indicated in the FokI-assisted signal amplification assay.

### Sample Collection and Detection of SARS-CoV-2 RNA Species in Human Samples

A total of 111 nasopharyngeal swab samples from patients subjected to COVID-19 PCR tests were collected at the Hospital Universitario Central de Asturias with the approval of the Research Ethics Committee of the Principality of Asturias (ref 2020.309). RNA was isolated using a MagNA pure 96 System (Roche Diagnostics) following the manufacturer’s recommendations. Detection of SARS-CoV-2 was performed via a coupled RCA—FokI-assisted signal amplification assay, as described in the previous section with some minor modifications: 4 μl of purified RNA was incubated in the presence of both Spike and Orf8a padlock probes in the padlock circularization step, and their corresponding mixture of 5′ FAM and 3′ BHQ-1 labeled dumbbell-like oligonucleotides in the simultaneous RCA/FokI-assisted digestion step. Detection of the virus was also performed using an in-house RT-PCR approach. Viral genomes were amplified using TaqMan Fast Virus 1-step Master Mix (Life Technologies, #4444432) and the CDC-recommended nucleoprotein-specific primers and VIC-labeled minor groove-binding (MGB) probes. Amplifications and data analysis were carried out in a StepOnePlus Real-Time PCR System under the following conditions: retrotranscription at 50 °C for 15 min, denaturation at 95 °C for 5 min, 40 cycles at 95 °C for 10 s (s), followed by 60 °C for 20 s.

## Results and Discussion

### Hairpin Guide Oligonucleotides Hijack Native FokI Activity in the Presence of DNA Duplexes and DNA/RNA Hybrids

**Figure 1:**
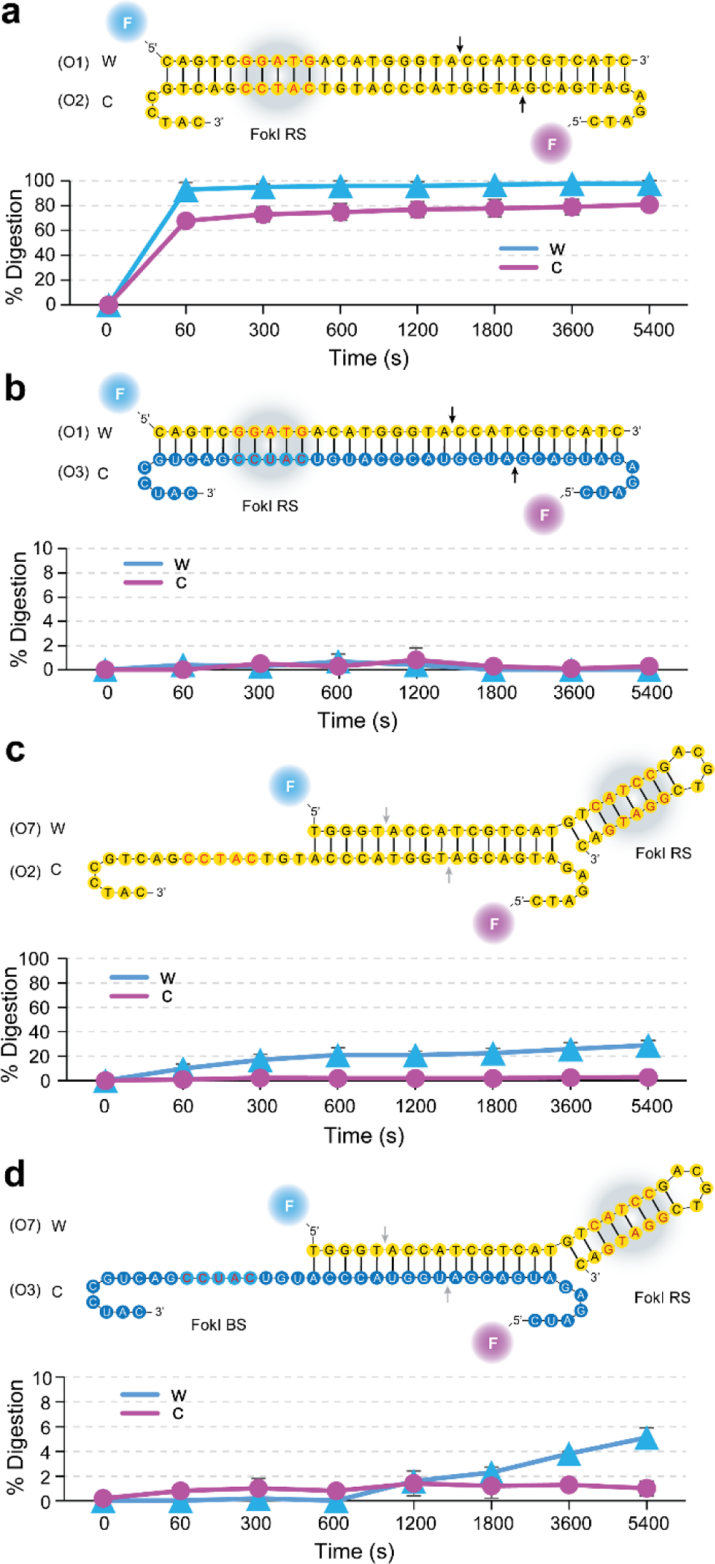
Modulation of FokI RE activity in DNA duplexes and DNA/RNA hybrids. (a) Sequence of DNA duplexes. (b) Sequence of DNA/RNA hybrids. (c) Sequence of the hairpin oligonucleotide probe with a complementary DNA strand. (d) Sequence of the hairpin oligonucleotide probe with a complementary RNA strand. For all conditions, experiments were done at least in duplicate, and the 5′ end labeling of the oligonucleotides with Cy5.5 (magenta) or IRD800 (blue) is indicated. W = Watson strand (blue), C = Crick strand (magenta). Yellow or blue dots denote the type of nucleic acid (DNA or RNA, respectively). The FokI binding site is indicated by the shaded region, red nucleotides indicate the RE recognition site, and arrows reflect the theoretical cleavage site. Measurements indicate the average percentage of digestion and the standard deviation of the W or the C strand, respectively.

Previous evidence has suggested that the catalytic domain of FokI might recognize and cleave the DNA strand in the context of an RNA/DNA heteroduplex substrate when fused to a given zinc finger motif.^14^ However, whether native FokI is able to retain this asymmetric cleavage capacity has not yet been adequately demonstrated. Thus, we designed short complementary DNA/DNA or DNA/RNA oligonucleotides labeled with either Cy5.5 or IRD800 dyes at their 5′ end and containing a single FokI restriction site (Figure 1). Because a previous work had demonstrated that BanI RE was able to digest both strands of a DNA/RNA hybrid,^13^ these sequences were designed to also include a single BanI restriction site as the internal control. FokI cleaved both substrate strands of a DNA duplex, and the cleavage occurred with high efficiency and at defined positions within the target sequence (Figures 1a, S1a). However, FokI-mediated cleavage of the heteroduplex DNA/RNA substrate did not yield any identifiable product even after long incubation times (Figures 1b and S1a). In contrast, the internal control reaction carried out with BanI RE resulted in the generation of defined restriction products (Figure S2a). To rule out any potential issues related to the star activity of the FokI RE, we generated new unrelated hybridization sequences that lacked the FokI restriction site (Figure S2b). The digestion of either DNA duplexes or DNA/RNA heteroduplexes by FokI RE was completely eradicated in the absence of a cognate restriction site, indicating that the cleavage reaction mediated by a native FokI is highly specific and depends on the presence of a DNA duplex and not a DNA/RNA hybrid in the recognition site itself.

To check whether the catalytic domain of FokI may still retain some activity in the absence of a suitable recognition site, we adopted a new strategy inspired by the structure of the hairpin guide oligonucleotides proposed by Weizmann and colleagues.^19^ These structures retain a double-stranded FokI recognition site in one of the oligonucleotides and have an overhang that can hybridize in a sequence-specific manner with additional complementary sequences. As FokI cleavage occurs 9–13 nt away from the recognition site, a virtual digestion of the cognate duplex may be achieved in the presence of such structures. We demonstrated that FokI-mediated digestion of a DNA duplex using this hairpin guide oligonucleotide yielded the desired restriction product in one of the strands (Figures 1c and S1b), although the efficiency of the reaction was reduced 3- to 4-fold (30% digested product) as compared with the kinetics of the native FokI endonuclease in the presence of a fully complementary substrate (Figure 1a). Interestingly, we did not observe any digestion product on the complementary strand, indicating that in the context of a DNA duplex, the hairpin guide oligonucleotide was able to digest itself in the presence of a complementary sequence but not in the absence of the complementary counterpart (Figure S2c). This result indicates that the RE activity of FokI may target unrelated trans sequences by modulating the hybridization properties of the guiding oligonucleotides. In the presence of a complementary RNA oligonucleotide (DNA/RNA hybrid), the hairpin guide oligonucleotide was able to induce a FokI relaxation (IRD), which resulted in the partial digestion of the hairpin guide oligonucleotide (Figures 1d and S1b). Despite the low efficiency of this process, the cleavage product was consistently and reproducibly detected (5% digested product). Together with previous observations using chimeric constructs between zinc finger domains and the catalytic domain of FokI,^14^ our results indicate that the catalytic activity of this RE can be uncoupled from its DNA-binding domain for the development of novel molecular strategies for the detection of nucleic acid targets.

### FokI-Assisted Digestion Enhances the Detection of DNA/RNA Heteroduplexes

The abovementioned results indicated that the use of hairpin guide oligonucleotides can hijack the cleavage activity of native FokI. We thus hypothesized that FokI-assisted digestion could enhance the detection of nucleic acids, either DNA or RNA, when these molecules are conjugated to a fluorescent dye–quencher pair. Figure S3 shows the details of the FokI-assisted reaction using a combination of a fluorescent dye and quencher molecules at the 5′ and 3′ends of the dumbbell-like structure in the context of a conventional hybridization assay, a basic FokI-assisted signal amplification assay, or an extended FokI-assisted signal amplification assay mediated by a DNA machine.^19^

**Figure 2:**
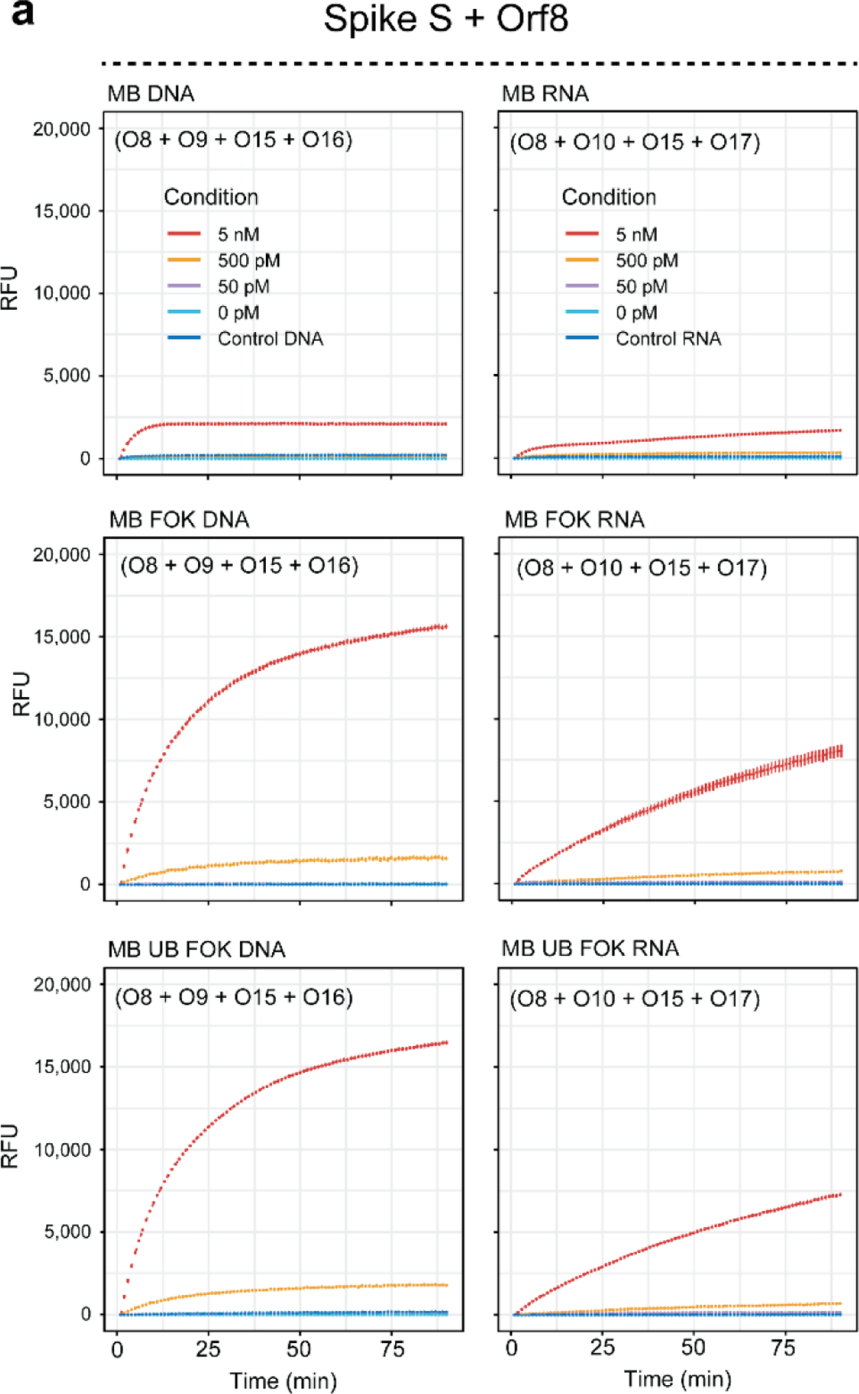
Enhanced detection of SARS-CoV-2 sequences by FokI-assisted digestion of DNA/RNA hybrids. (a) Line plots displaying the real-time fluorescence detection measurements of a conventional hybridization assay (upper panel), a basic FokI-assisted signal amplification assay (middle panel), and an extended FokI-assisted signal amplification assay in the presence of universal hairpin oligonucleotides (lower panel). Different concentrations (0–5 nM) of DNA (left panels) or RNA (right panels) substrates corresponding to combined Spike and Orf8 sequences were assayed. Unrelated DNA or RNA sequence was used at a constant concentration of 50 nM for control purposes. For all the experiments, the concentration of the reporter dumbbell-like oligonucleotides was kept constant at 100 nM. Lines represent the average signal detection at a given time point, and error bars indicate the standard deviation of 2 independent experiments performed in duplicate (*n* = 4).

In order to test the efficacy of the proposed signal amplification methods for the detection of real-world pathogens, we focused on the study of the recently isolated human Beta coronavirus SARS-CoV-2. The adopted strategy led to the design of 3 dumbbell-like oligonucleotides, labeled with 6-FAM and BHQ1 at their 5′ and 3′ ends, respectively, and of their complementary synthetic DNA and RNA substrates, each corresponding to the coding regions of the Nsp4, Spike, and Orf8 proteins of SARS-CoV-2 (Figure S4a). We then examined the performance of the proposed signal amplification strategies in the presence of different DNA or RNA target concentrations. The extent of the signal amplification was sequence dependent, and the dumbbell-like oligonucleotide designed against the Spike region resulted in the greatest improvement in fluorescence signal amplification when coupled with FokI-assisted digestion, as compared to conventional hybridization assays (Figures S5 and S6). In addition, the simultaneous combination of multiple dumbbell-like oligonucleotides and target sequences (Spike and Orf8a) in the same reaction mixture resulted in a further enhancement of the detection signal (Figure 2) as compared to the aforementioned singleplexed reactions (Figure S6). In fact, we observed that regardless of the target context, the presence of the universal hairpin beacon oligonucleotide slightly improved the detection of the fluorescence signal, particularly in the case of RNA molecules. Although the performance of the reaction was better in the presence of DNA substrates, the improved detection of RNA molecules was also enhanced by both the basic and the extended FokI-assisted signal amplification assays. Indeed, for the case of the combined Spike and Orf8 regions, the limit of detection (LOD) was improved more than 3.5-fold in the case of DNA and 3-fold for RNA targets (Figure S5, LOD conventional assay DNA/RNA = 0.6 and 0.91 nM, LOD basic FokI-assisted digestion DNA/RNA = 0.16 and 0.27 nM, LOD extended FokI-assisted digestion DNA/RNA = 0.13 and 0.20 nM, respectively).

### Specificity and Multiplex Capabilities of the FokI-Assisted Signal Amplification Assay

**Figure 3:**
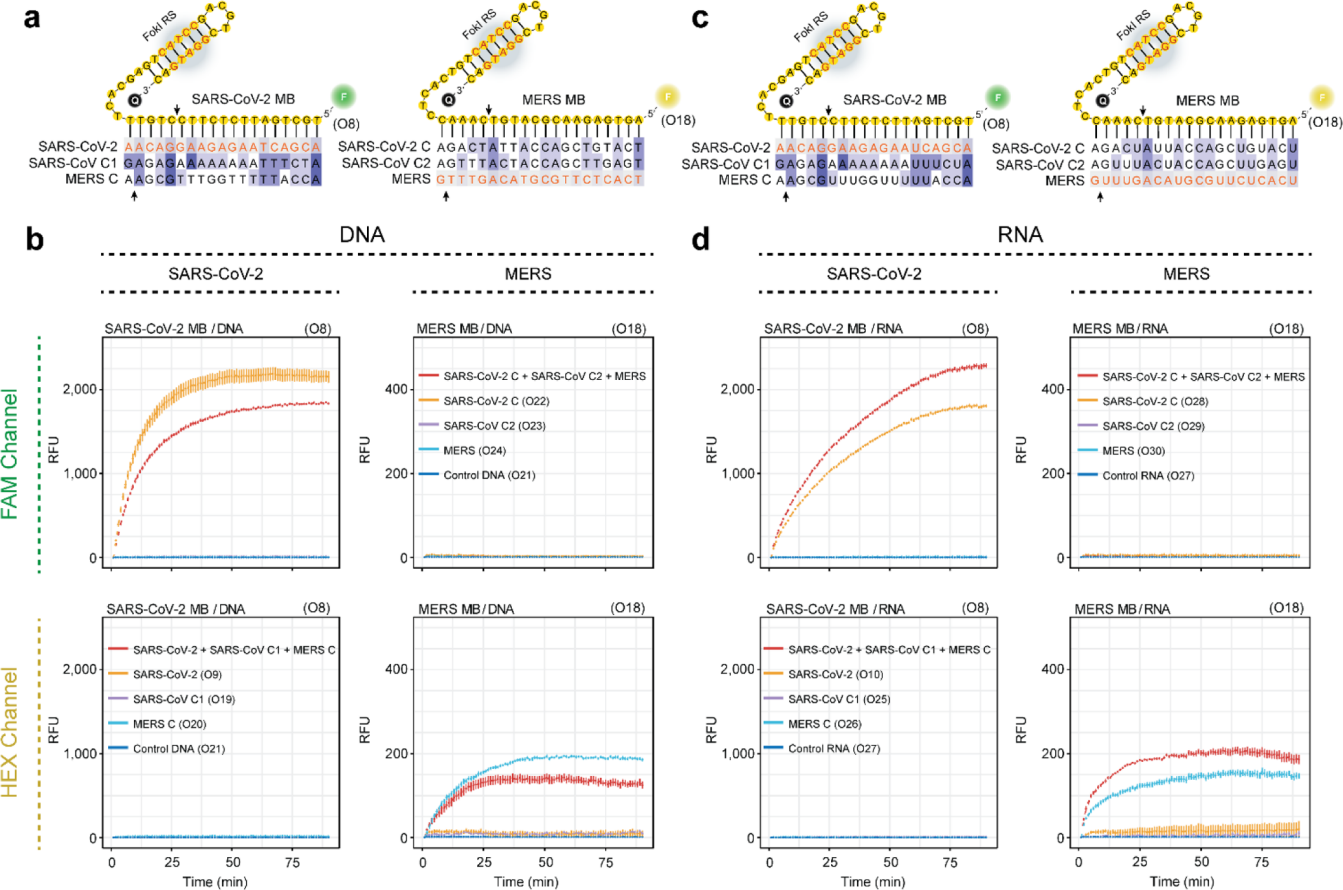
Specificity of the FokI-assisted signal amplification reaction. (a) Schema depicting the DNA sequence and the multiple sequence alignment of the human Beta coronaviruses SARS-CoV-2, SARS-CoV, and MERS interrogated in these experiments (green = 6-FAM, yellow = HEX, Q = BHQ1 quencher). (b) Plots illustrating the real-time fluorescence detection measurements of the basic FokI-assisted signal amplification assay simultaneously recorded in the FAM and HEX channels. Experiments were performed at a constant concentration of 5 nM for each of the substrates indicated in the legend, with the exception of the control sequence, which was assayed at a concentration of 50 nM for control purposes. For all the experiments, the concentration of each reporter dumbbell-like oligonucleotide was kept at a constant concentration of 100 nM. Lines represent the averaged signal and the standard deviation of 2 independent experiments performed in duplicate (*n* = 4). (c,d) Same as (a,b) but in the context of DNA/RNA hybrids.

To test the specificity of the system in distinguishing SARS-CoV-2 targets from divergent orthologous sequences, we performed a similar set of experiments in the presence of complex DNA or RNA mixtures from various human Beta coronaviruses. We focused on the dumbbell-like oligonucleotide designed against the Spike region of SARS-CoV-2 as this construct achieved the best performance in terms of signal amplification. We also designed an additional dumbbell-like oligonucleotide specific to the Spike region of MERS (Figure S4b). To avoid signal interference, the latter construct was labeled with HEX fluorophore at its 5′ end. These custom dumbbell-like structures displayed a perfect sequence homology with SARS-CoV-2 and MERS but contained a number of mismatches compared to other orthologous sequences of the SARS-CoV and MERS human coronaviruses (Figure 3a,b, S4), which were included for control purposes.

The enhanced detection of SARS-CoV-2 sequence was achieved only in the presence of the corresponding DNA or RNA target molecule and the custom dumbbell-like oligonucleotide in the FAM channel (Figure 3b,d, upper left panel) but not in the presence of other SARS-CoV-2-related sequences, corresponding to the orthologous sequences of SARS-CoV, MERS, or unrelated control substrates. In addition, no signal was detected in the HEX channel (Figure 3b,d, lower left panel), indicating that the reporter dumbbell-like oligonucleotide efficiently exerts its fluorescence in the corresponding emission spectra. Conversely, the detection of MERS by its corresponding dumbbell-like oligonucleotide was equally well achieved regardless of the complexity of the DNA or RNA mixture, and the detection of MERS and no other related orthologous sequences (SARS-CoV, SARS-CoV-2) was obtained in the range of the HEX channel (Figure 3b,d, bottom right). These results were observed in the presence of complementary DNA or RNA substrates and indicate that the amplification mediated by FokI cleavage is highly specific regardless of the complexity of the samples.

Given the specificity of the previous reaction, we hypothesized that a combination of custom dumbbell-like structures and their corresponding nucleic acid substrates could be used to detect multiple targets simultaneously. Thus, we combined in the same reaction the aforementioned dumbbell-like oligonucleotides that target SARS-CoV-2 or MERS, which were labeled with 6-FAM or HEX, respectively, and a mixture of DNA or RNA substrates including either SARS-CoV-2, MERS, or a combination of both synthetic targets (Figure S7a,b, respectively, for DNA duplex or DNA/RNA hybrid detection). The detection of each single DNA or RNA condition of SARS-CoV-2 or MERS (red lines) was achieved only in the presence of the corresponding fluorescent channel (FAM or HEX, respectively), despite the reaction mixture containing both reporter dumbbell-like oligonucleotides (Figure S7a-c). The same was also observed in complex nucleic acid mixtures that included the individual DNA or RNA condition of SARS-CoV-2 or MERS in the presence of other related orthologous control sequences (yellow lines), indicating that this system can discriminate their complementary nucleic acids regardless of the complexity of the sample. Interestingly, samples containing both SARS-CoV-2 and MERS sequences (purple lines) were successfully detected in both fluorescent channels, this dual detection being similarly achieved in the presence of either DNA or RNA substrates (Figure S7a,b respectively), thereby validating the multiplexing potential of the FokI-assisted signal amplification assay.

### FokI-Assisted Signal Amplification Can Discriminate between Currently Known SARS-CoV-2 Variants

To test whether the FokI-assisted signal amplification assay was specific enough to discriminate between recently described SARS-CoV-2 variants, which often differ by only a single nucleotide, we designed novel reporter dumbbell-like structures that were specific to either the wild-type or the B.1.1.7 strains (labeled with FAM or HEX at their 5′ end, respectively) and their corresponding synthetic DNA target sequences in the vicinity of the Spike N501Y mutation (Figure S8a).

When these reactions were performed with their specific, single dumbbell-like structures, signal amplification was achieved with both wild-type and B.1.1.7 target molecules, but not with control sequences, in their corresponding FAM and HEX fluorescent channels (Figure S8b, left and middle panels), although the signal intensity of their fully complementary cognate molecules always outperformed that detected in the context of single nucleotide mismatches. We therefore tested whether the multiplex addition of both dumbbell-like oligonucleotides in the same reaction could compete with and enhance the detection of a given target molecule. In addition to the fact that the simultaneous presence of both fluorescent-labeled dumbbell-like structures still resulted in both wild-type and mutant target molecules being detected, we also observed an improvement in the discrimination of the target sequences in their corresponding channels (Figure S8b, right panel) as compared to the singleplex scenario (Figure S8b, left and middle panels).The multiplex combination of FAM and HEX signal trajectories in a given sample, calculated by means of the principal component analysis, displayed a clear, time-dependent divergence between the two sequence variants (Figure S8c), indicating that the combination of multiple reporter dumbbell-like structures specific to the different SARS-CoV-2 variants in a single mixture can facilitate the discrimination of SARS-CoV-2 strains with minimal sequence divergence.

### Improved Detection of Target Molecules in the Context of Coupled FokI-Assisted Signal Amplification and Isothermal Amplification of Nucleic Acids

In order to assay whether our in vitro model could be applied to detecting and discriminating between human SARS-CoV-2 variants, we tested the efficacy of our proposed detection method in RNA isolated from nasopharyngeal swab samples. Unfortunately, our pilot experiments with patient samples did not render any positive results (data not shown), indicating that the limit of detection of the basic FokI-assisted assay was out of the detection range, as has recently been observed in human samples.^20^ Since the start of the COVID-19 pandemic, several studies have reported the use of alternative nucleic acid detection technologies, based on either RT-LAMP,^21^ recombinase polymerase amplification,^22^ or a combination of the CRISPR/Cas12a/13 system^23−26^ with any of the aforementioned techniques, for the rapid monitoring of SARS-CoV-2 infection. Despite the usefulness of these approaches, most of these reactions require the use of a reverse transcriptase to detect the viral RNA target. Thus, we decided to overcome this limitation and improve the detection limit of our assay by considering a simultaneous molecular coupling of the FokI-assisted reaction and the RCA technique,^27,28^ which relies on the isothermal amplification of circular DNA targets mediated by the high processivity and strand displacement activity of the Phi29 DNA polymerase.^29−31^

**Figure 4:**
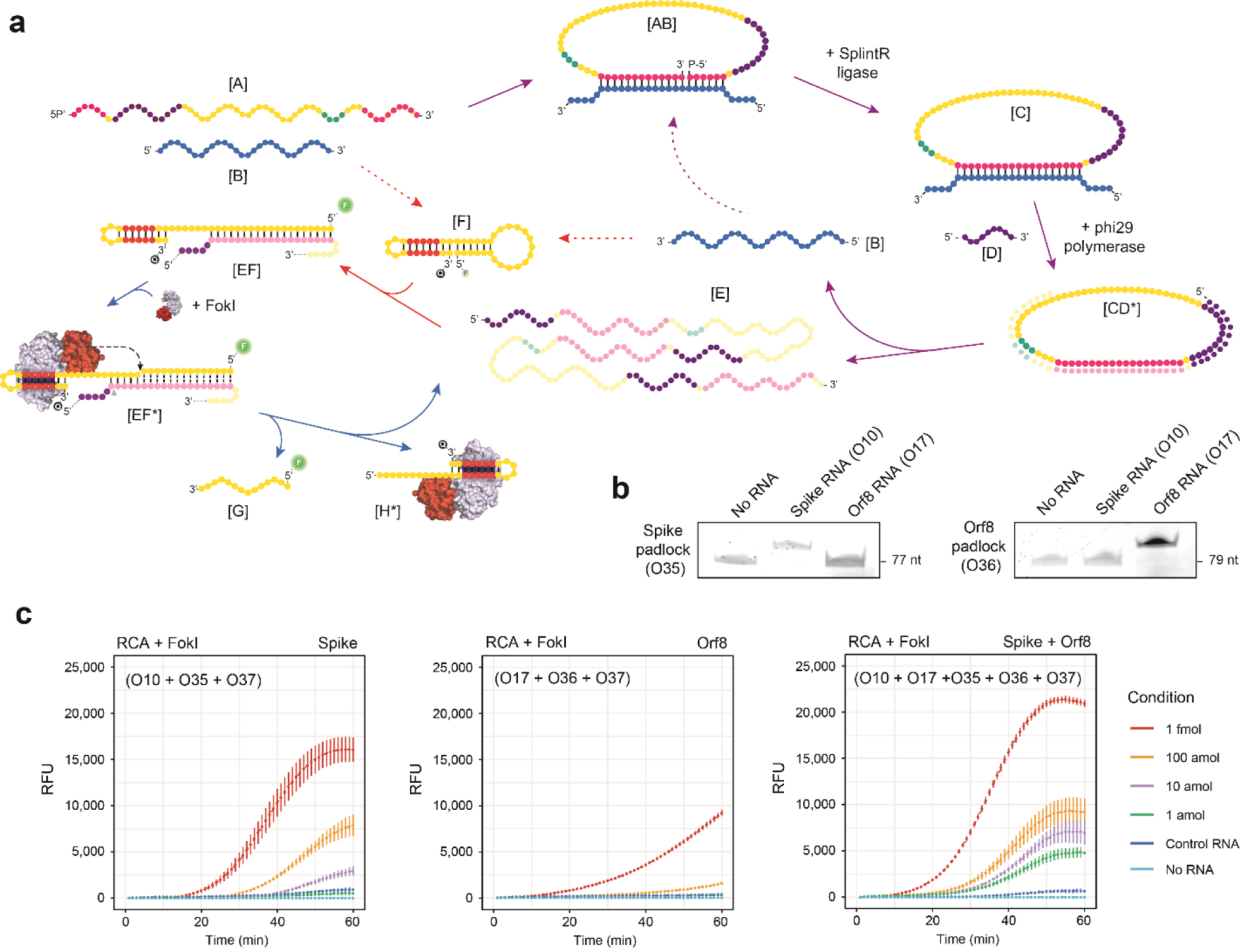
Molecular coupling between FokI-assisted signal amplification and RCA. (a) Schema depicting the different stages of the RCA reaction and the molecular coupling with the FokI-assisted signal amplification method. Purple arrows: classical RCA reaction. Red arrows: conventional hybridization assay. Blue arrows: basic FokI signal amplification assay. (b) Graphs indicate the results of the ligation assay including Spike or Orf8 padlock probes and their complementary target RNAs. The indicated oligonucleotides (250 nM each) were incubated with 12.5 units of SplintR ligase for 15 min at room temperature, and ligation products were run on 12% denaturing PAGE gels for visualization purposes. (c) Plots illustrating the real-time fluorescence detection measurements of an RCA reaction coupled with the FokI-assisted signal amplification system in the presence of dumbbell-like oligonucleotides against Spike (left panel), Orf8a (middle panel), or both reporter constructs (right panel). Different amounts of target RNA substrates (1 fmol, 100, 10, 1 amol) corresponding to individual or combined sequences of SARS-CoV-2 Spike and Orf8 regions were assayed for 60 min, and 1 fmol of an unrelated RNA sequence was used for control purposes. The concentration of the reporter dumbbell-like oligonucleotides was kept at a constant concentration of 100 nM. Lines represent the averaged signal and the standard deviation of 3 independent experiments.

Figure 4a shows the details of the RCA reaction, which involves the use of padlock probes [A] and a cognate nucleic acid target [B]. The hybridization of a target molecule with a padlock probe reorganizes the configuration of the padlock probe, which adopts a circular structure [AB]. The catalytic activity of a DNA ligase enzyme facilitates the full circularization of the padlock probe by ligating its splinted 5′ and 3′ ends [C]. The resulting single-stranded DNA template can be used for indefinite rounds of amplification in the presence of a complementary RCA primer [D], dNTPs, and the DNA polymerase Phi29, thus increasing the number of target sequences in the reaction mixture [E]. Within this context, we hypothesized that the simultaneous molecular coupling of the FokI-assisted signal amplification assay might improve the detection results obtained in classical RCA approaches. Target molecules [B, E] can hybridize with the custom-designed dumbbell structures [F] and generate a duplex/heteroduplex substrate [BF, EF] that can release the fluorescence signal of the reporter molecule. The asymmetric cleavage reaction mediated by FokI can enhance the excision of the fluorescent portion of the probe [G] and the potential reuse of the target sequence [B, E] for further rounds of digestion, thus improving the detection signal of reaction.

To test the efficiency of the padlock probe circularization approach for the detection of SARS-CoV-2 sequences, we designed custom DNA padlock oligonucleotides that complemented the Spike and Orf8a regions of SARS-CoV-2 in the vicinity of the sequences designed for the dumbbell-like oligonucleotides (Figure S9a). Incubation of these padlock probes with their RNA targets and the SplintR DNA ligase led to successful circularization in the presence of their corresponding target molecules but not with unrelated RNA sequences (Figure 4b). The specificity of the Spike padlock probes was further confirmed using RNA sequences corresponding to the SARS-CoV-2 Spike region or orthologous RNAs from closely related coronaviruses (SARS-CoV, MERS, Figure S9a,b). The subsequent molecular coupling of FokI-assisted digestion and the RCA reaction at short incubation times (<60 min) enhanced the detection limit of the basic FokI-assisted signal amplification assay by 2 to 4 orders of magnitude depending on the target RNA sequence used (Orf8a or Spike), with the dumbbell-like oligonucleotide designed against the Spike region being the construct that displayed the greatest improvement in fluorescence signal amplification (Figure 4c). The presence of the FokI endonuclease in the reaction mixture greatly outperformed the response of the fluorescence signal alone and reduced the detection time as compared with a classical RCA approach. In fact, the absence of the endonuclease did not induce tangible changes in FAM fluorescence between conditions, even after 120 min of incubation time (Figure S9c). Interestingly, the simultaneous combination of both dumbbell-like oligonucleotides and target sequences (Spike and Orf8a) also resulted in an additive enhancement of the detection signal (Figure 4c, right panel) as compared to that of singleplex reactions. Under these conditions, we achieved a theoretical limit of detection of SARS-CoV-2 RNA of 2.8 × 10^5^ molecules per mL (Figure S9d), a level resembling the conditions observed in the clinical practice.^20^

### Detection of SARS-CoV-2 RNA in Human Samples

**Figure 5:**
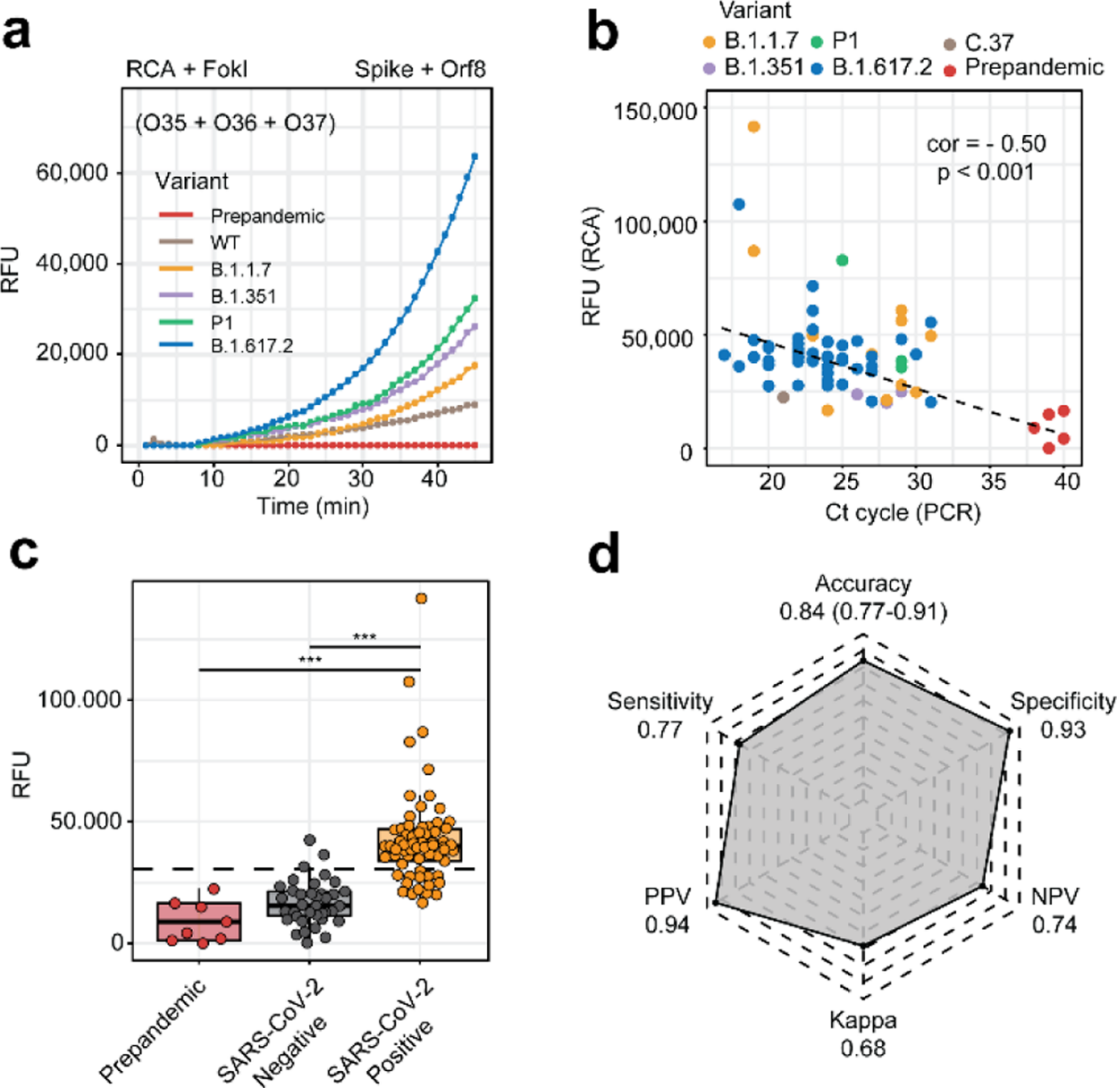
Detection of SARS-CoV-2 in samples from COVID-19 patients. (a) Plots showing the real-time fluorescence detection measurements of an RCA reaction coupled with the FokI-assisted signal amplification system. Reactions were monitored for 45 min using samples from COVID-19 patients comprising different SARS-CoV-2 variants (WT, B.1.1.7, B.1.351, P.1, and B.1.617.2). A pre-pandemic sample was used for control purposes, and the concentration of both reporter dumbbell-like oligonucleotides was kept at a constant concentration of 100 nM. (b) Plot reflecting the relationship between RT-qPCR (*x*-axis) and FokI-assisted RCA results in the positive samples. (c) Plots showing the relative fluorescence units of SARS-CoV-2-positive or control cases as obtained from the previous coupled assays. Dashed line indicates the relative fluorescence corresponding to the z-score used for statistical purposes (*** = *p*-value < 0.001, one-tailed Welch’s *T*-test). (d) Radar plots illustrate the metrics and performance of the FokI-assisted signal amplification method as compared to the gold standard qRT-PCR approach.

Inspired by these results, we further interrogated whether this molecular coupling could be used as a diagnostic method for the detection of SARS-CoV-2 particles in RNA from human samples. We initially tested whether our original dumbbell-like structures were able to identify the presence of viral sequences in the context of multiple SARS-CoV-2 variants isolated from nasopharyngeal swabs, including the WT, B.1.1.7, B.1.351, P.1, and B.1.617.2 strains, as well as other pre-pandemic known human coronaviruses (Figure 5a). Interestingly, we achieved a successful detection of SARS-CoV-2 RNA irrespective of the type of variant interrogated, and such signal amplification was not detected in pre-pandemic controls, indicating that the designed dumbbell-like structures were highly specific for SARS-CoV-2 and resilient to recently identified mutations.

We further extended this analysis to a cohort of 46 control samples and 65 diagnosed COVID-19 cases that included different variants of interest. The results indicated a significant anti-correlation between the signal intensity of positive SARS-CoV-2 cases obtained in these FokI-assisted signal amplification assays and sample cycle thresholds obtained by RT-PCR approaches (Figure 5b). Significant differences in the signal intensity of SARS-CoV-2-positive and negative cases were observed at reaction times of 45 min (Figure 5c), and the accuracy, sensitivity, and specificity of the FokI-assisted signal amplification reaction as compared to the gold standard RT-PCR approach were, respectively, 0.84, 0.77, and 0.93 (Figure 5d).

## Discussion

The method proposed in this work reveals the unanticipated activity exerted by the type IIS endonuclease FokI in the context of DNA duplexes and DNA/RNA hybrids and highlights the possibility of considering this reaction as a potential diagnostic method for the identification of nucleic acids of particular relevance for human health. An important advantage of this approach relied on its high specificity toward their cognate molecular targets. In addition, the combination of multiple dumbbell-like oligonucleotides labeled with different fluorophores in a multiplex assay allowed for the simultaneous detection of multiple sequences, that is, the specific discrimination of particular SARS-CoV-2 variants. Lastly, the simultaneous molecular coupling between the FokI-assisted signal amplification assay and the RCA technology significantly improved the diagnostic capacity of the reaction as compared to classical RCA approaches, resulting in high specificity and sensitivity and shorter detection times, demonstrating the high versatility and cost-effectiveness of the FokI-assisted signal amplification technology for the detection of viral pathogens.

## Supporting Information Available

List of sequences used in this work, FokI-assisted digestion of DNA duplexes and DNA/RNA hybrids, digestion of DNA duplexes and DNA/RNA hybrids mediated by BanI and FokI, working principle of the FokI-assisted digestion method, design of target DNA/RNA sequences and reporter oligonucleotides, LOD of the proposed reactions, enhanced detection of individual SARS-CoV-2 sequences, FokI-assisted signal amplification allows the simultaneous detection of multiple targets, FokI-assisted signal amplification discriminates between known SARS-CoV-2 variants, and specificity and LOD of the RCA-enhanced reaction (PDF)
