# Supplementary material for: Enhanced Detection of Viral RNA Species Using FokI-Assisted Digestion of DNA Duplexes and DNA/RNA Hybrids
Source: Anal Chem. 2022 Apr 25;94(18):6760–70. doi: 10.1021/acs.analchem.2c00407 (PMC9063116; doi:10.1021/acs.analchem.2c00407)
Supplement: Supplementary file 1 — ac2c00407_si_001.pdf [file ac2c00407_si_001.pdf]

Supporting Information

Enhanced detection of viral RNA species using FokI-assisted digestion of DNA duplexes and DNA-RNA hybrids

Juan R. Tejedor[1, 2, 3, 4, 6, *], Gabriel Martín[5, 6], Annalisa Roberti[1, 2, 6], Cristina Mangas[2, 3, 6], Pablo Santamarina-Ojeda[2, 3, 6], Raúl F. Pérez[1, 2, 3, 6], Virginia López[2, 3, 6], Rocío G. Urdinguio[2, 4, 6], Juan J. Alba-Linares[1, 2, 6], Alfonso Peñarroya[1, 2, 6], Marta E. Álvarez-Argüelles[5, 6], José A. Boga[5, 6], Agustín F. Fernández[1, 2, 3, 4, 6], Susana Rojo-Alba[5, 6] and Mario F. Fraga[1, 2, 3, 4, 6, *]

1.Nanomaterials and Nanotechnology Research Center (CINN-CSIC), El Entrego, 33940, Spain.

2.Foundation for Biomedical Research and Innovation in Asturias (FINBA), Oviedo, 33011, Spain.

3.University Institute of Oncology (IUOPA), University of Oviedo, Oviedo, 33006, Spain.

4.Center for Biomedical Network Research on Rare Diseases (CIBERER), Madrid, 28029, Spain.

5.Central University Hospital of Asturias (HUCA) Oviedo, 33011, Spain.

6.Health Research Institute of Asturias (ISPA), Oviedo, 33011, Spain.

* To whom correspondence should be addressed.

Juan Ramón Tejedor: Avenida del Hospital Universitario s/n 33011 Oviedo. Asturias. España ; +34 985733644; jr.tejedor@cinn.es

Mario F. Fraga: Avenida del Hospital Universitario s/n 33011 Oviedo. Asturias. España ; +34 985733644; mffraga@cinn.es

Table of contents

Supplemental Materials and Methods S2
Strand-specific analysis of FokI / BanI endonuclease activity. S2
Design of synthetic oligonucleotides against human coronaviruses S2
Specificity assays S3
Data normalization and batch correction S4
Supplemental Table and Figure legends S6
- Table S1. List of sequences used in this work S6
- Figure S1. FokI-assisted digestion of DNA duplexes and DNA:RNA hybrids (related to Figure 1).. .... S7
- Figure S2. Digestion of DNA duplexes and DNA:RNA hybrids mediated by BanI and FokI S8
- Figure S3. Working principle of FokI-assisted digestion method S10
- Figure S4. Design of target DNA/RNA sequences and reporter oligonucleotides S12
- Figure S5. LOD of the different reactions tested S13
- Figure S6. Enhanced detection of individual SARS-CoV-2 sequences S14
- Figure S7. FokI-assisted signal amplification allows the simultaneous detection of multiple targets S15
- Figure S8. FokI-assisted signal amplification discriminates between known SARS-CoV-2 variants S16
- Figure S9. Specificity and LOD of the RCA enhanced reaction S17
Supplemental References S18

Supplemental Materials and Methods

Strand-specific analysis of FokI / BanI endonuclease activity.

Samples including RNA and DNA oligonucleotides were denatured at 95ºC for 2 min and left at room temperature for 5 min to achieve full annealing between complementary oligonucleotides. Restriction enzyme digestions were performed at 37ºC for 1, 5, 10, 20, 30, 60 and 90 min in a reaction mix comprising 2 µl of NEB 10X Cutsmart buffer (NEB #B7204, 1X Buffer composition: 50 mM Potassium Acetate, 20 mM Tris-Acetate, 10 mM Magnesium Acetate, 100 µg/ml BSA, pH 7.9 at 25ºC), 50nM (1 pmol) of the duplex or heteroduplex substrate and either 5 units of the RE FokI (NEB, #R0109S) or 10 units of BanI (NEB, #R0118S) in a final volume of 20 µl. Reaction products were mixed with one volume of 2X denaturing gel-loading urea buffer (90 mM Tris, 90 mM Boric acid, 2 mM EDTA, pH 8, 12% glycerol, 0.01% Bromo-phenol blue, 7 M Urea) and heated to 95ºC for 2 min. 20 µl aliquots were resolved by denaturing polyacrylamide gel electrophoresis in 15% polyacrylamide/urea/TBE denaturing precast gels (BioRad, #4566053). IRD800 and Cy5.5 fluorescence corresponding to the different oligonucleotides was monitored by gel scanning using a LI-COR Fc Odyssey variable mode imager (LI-COR biosciences). Excitation wavelengths of 685nm (with 700nm channel) and 785nm (with 800nm channel) were used for Cy5.5 and IRD800 scans, respectively. Densitometric analyses of the corresponding bands were performed with ImageStudio software (LI-COR biosciences).

Design of synthetic oligonucleotides against human coronaviruses

The FASTA sequences corresponding to 7 human infectious coronaviruses (SARS-CoV-2, SARS-CoV, MERS, HKU1, OC43, HCoV-229E and NL63) were downloaded from NCBI (accession numbers NC_045512.2, NC_004718.3, KT121573, KF686346.1, AY391777.1, KU291448.1, JX504050.1, respectively). Multiple sequence alignment was performed with the computational tool Clustal Omega[1] implemented in the European Bioinformatics Institute (EBI) servers using as input the aforementioned cDNA sequences. Visualization of the multiple sequence alignments was performed with the Jalview software (v2.11)[2]. For the identification SARS-CoV-2 sequences with common hybridization regions compatible with custom-designed dumbbell-like oligonucleotides, we initially scanned the occurrence of all possible combinations of 6-mer along the reverse complement SARS-CoV-2 genome with the out-of-core k-mer counter Meryl (rev2008)[3]. Manual inspection of sequences surrounding the TGCTGA hexamer was used to explore regions with maximum number of mismatches between the different human Beta coronaviruses. Among the resulting candidates, we selected those sequences that were characterized for the presence of 6 bp in the stem structure of the dumbbell-like oligonucleotide in order to avoid self-cleavage by FokI prior to the hybridization step. The results of this top-down approach led to the design of custom dumbbell-like oligonucleotides against 3 regions of the SARS-CoV-2 genome, corresponding to the open reading frames of the Spike, Nsp4 and Orf8 proteins. The aforementioned oligonucleotides were labelled with 6-FAM fluorophore and BHQ-1 quencher at their 5' and 3' ends, respectively, and these sequences were characterized by a common hairpin region including a unique FokI restriction site. Dumbbell-like structures were confirmed in silico using the RNAstructure software[4]. For evaluation purposes, we designed short synthetic RNAs and DNAs corresponding to the aforementioned SARS-CoV-2 genomic regions or to a random oligonucleotide sequence (30 nt length) in order to set up the FokI-assisted signal amplification reactions. To enhance the amplification of the fluorescent signal by the introduction of feedback loops, we also designed a universal hairpin oligonucleotide capable of hybridizing with the reaction product of any of the previous RE reactions. This universal hairpin was labelled with 6-FAM fluorophore and BHQ-1 quencher at its 5' and 3' ends, respectively, and its structure reassembled the DNA machine construct proposed by Weizmann et al.

Specificity assays

To test the specificity of the FokI-assisted signal amplification assay, different combinations of DNA or RNA oligonucleotides corresponding to orthologous sequences of SARS-CoV-2, SARS-CoV or MERS coronaviruses were tested against different custom dumbbell-like oligonucleotides. Specificity for SARS-CoV-2 was assayed using the Spike dumbbell-like oligonucleotide and related DNA/RNA sequences as for in the basic FokI-assisted signal amplification assay. In addition, a second dumbbell-like oligonucleotide, complementary to MERS Spike protein, but not to other human Beta coronaviruses, was designed and labelled with HEX fluorophore and BHQ-1 quencher, respectively, at its 5' and 3' end. Other dumbbell-like oligonucleotides, complementary to the wild-type (N501) or the alpha (N501Y) SARS-CoV-2 variants, were also designed and labelled with FAM and HEX fluorophores at their 5' and 3' ends, respectively. All reactions were performed in a final reaction volume of 20 µl and at a constant temperature of 37ºC. The final concentrations of the reaction components were 2 µl of NEB 10X

Cutsmart buffer (NEB #B7204), 5 units of FokI RE (NEB, #R0109S), 50 nM of custom dumbbell-like oligonucleotide complementary to either SARS-CoV-2 variants or MERS synthetic regions, and 5 nM of each target DNA, target RNA or mock sequences in each corresponding reaction, with the exception of the Control DNA/RNA, which was assayed at 50 nM (10-fold excess). The different reactions were followed by the recording over time of the fluorescence intensity, as mentioned above, but the excitation and emission wavelengths were configured to detect FAM and VIC channels simultaneously (488 nm excitation, 520 nm emission, and 529 nm excitation, 549 nm emission, respectively). HEX dye has an excitation/emission spectrum similar to VIC dye (538 nm excitation and 554 nm emission). In the case of the multiplexed reactions, a similar set of experiments was performed. However, these reactions simultaneously incorporated 50 nM of SARS-CoV-2 / MERS or SARS-CoV-2 / SARS-CoV-2 variants custom dumbbell-like oligonucleotides, labelled with FAM and HEX fluorophores respectively.

Data normalization and batch correction

Basic FokI-assisted signal amplification assays: Multicomponent data were downloaded from the instrument and raw data from independent experiments were normalized for potential batch effects using the ComBat function of the R/Bioconductor package sva[5]. To facilitate comparisons between the different methodologies, and to avoid potential confounding effects caused by the different levels of background signals, signal intensities for the different conditions at the different time points were firstly normalized to the signal of each corresponding sample at time 0 (intra-sample normalization), and then to the signal of the non-template control (inter-sample normalization) by an iterative subtraction of the average intensities obtained at 0 M condition in each of the cycles of the experiment according to the following formula:

$$\boldsymbol{SI_{(t)} = X_{(t)} - \sum_{i=1}^{n} \frac{C_{i,(t)}}{n}}$$

where X(t) represents the signal intensity of a particular condition at a given time point t, Ci,(t) represents the signal intensity of the non-template control (0 M) at the same time point t, and n represents the number of replicates performed at the aforementioned time point t. The limit of detection at the end of the reaction was calculated as:

$$\boldsymbol{LOD = 3.3\,\frac{S_y}{Slope}}$$

where Sy represents the standard error of the predicted y-value for each x in the regression and Slope represents the slope value (a) of the calibration plot y= ax + b. The estimation of Sy was performed with the STEYX function according to the following formula:

$$\mathbf{S_y} = \sqrt{\frac{\mathbf{1}}{(\mathbf{n-2})}\left[\sum(\mathbf{y}-\bar{\mathbf{y}})^2 - \frac{[\sum(\mathbf{x}-\bar{\mathbf{x}})(\mathbf{y}-\bar{\mathbf{y}})]^2}{\sum(\mathbf{x}-\bar{\mathbf{x}})^2}\right]}$$

where x and y are the sample means average (known_x's and known_y's), and n is the sample size.

Combined reaction RCA – FokI-assisted signal amplification assays using human samples: Per-sample background normalization was performed by correcting the signal intensities at the different time points against the signal of each corresponding sample at time 0. For each sample, standard scores (z-scores) were calculated according to the normalized fluorescence intensity obtained from 9 validated pre-pandemic negative control cases at a given time point. Samples with z-scores > 2.5 ($p < 0.01$) were considered to be SARS-CoV-2 positive for viral identification purposes.

Supplemental Table and Figure legends

Name	Code	5' Mod	3' Mod	Sequence
DNA 1 Watson	O1	IRD800	-	CAGTCGGATGACATGGGTACCATCGTCATC
DNA 1 Crick	O2	Cy5.5	-	CTAGAGATGACGATGGTACCCATGTCATCCGACTGCCTAC
RNA 1 Crick	O3	Cy5.5	-	CUAGAGAUGACGAUGGUACCCAUGUCAUCCGACUGCCUAC
DNA 2 Watson	O4	IRD800	-	GTCAGCCTACTGTACCCATGGTAGCAGTAG
DNA 2 Crick	O5	Cy5.5	-	GATCTCTACTGCTACCATGGGTACAGTAGGCTGACGGATG
RNA 2 Crick	O6	Cy5.5	-	GAUCUCUACUGCUACCAUGGGUACAGUAGGCUGACGGAUG
DNA3 Watson	O7	IRD800	-	TGGGTACCATCGTCATGTCATCCGACGTCGGATGAC
MB1 SARSCOV2 (Spike)	O8	FAM	BHQ1	TGCTGATTCTCTTCCTGTTTCAGCAGTCATCCGACGTCGGATGAC
DNA1 SARSCOV2 (Spike)	O9	-	-	CTTGGAACAGGAAGAGAATCAGCAACTGTG
RNA1 SARSCOV2 (Spike)	O10	-	-	CUUGGAACAGGAAGAGAAUCAGCAACUGUG
Fuel MB1	O11	FAM	BHQ1	TGCTGAGCAGCCTCCATCCACTCTGCGCAGAGTGGATGGAGGCTGC
MB2 SARSCOV2 (Nsp4)	O12	FAM	BHQ1	TGCTGATATGTCCAAAGTCAGCAGTCATCCGACGTCGGATGAC
DNA2 SARSCOV2 (Nsp4)	O13	-	-	ATTGGTGCTTTGGACATATCAGCATCTATA
RNA2 SARSCOV2 (Nsp4)	O14	-	-	AUUGGUGCUUUGGACAUAUCAGCAUCUAUA
MB3 SARSCOV2 (ORF8)	O15	FAM	BHQ1	TGCTGATTTTCTAGCTCCTTCAGCAGTCATCCGACGTCGGATGAC
DNA3 SARSCOV2 (ORF8)	O16	-	-	AGAGTAGGAGCTAGAAAATCAGCACCTTTA
RNA3 SARSCOV2 (ORF8)	O17	-	-	AGAGUAGGAGCUAGAAAAUCAGCACCUUUA
MB1 MERS (Orf5)	O18	HEX	BHQ1	AGTGAGAACGCATGTCAAACCTCACTGTCATCCGACGTCGGATGAC
Control1 DNA SARSCOV	O19	-	-	CATGGGAGAGAAAAAAAATTTCTAATTGTG
Control1 DNA MERS	O20	-	-	ATTTCAAGCGTTTGGTTTTTACCAATTGCA
Control DNA	O21	-	-	TGCTAGAAAACCGCGTTTCTACGACTGGTG
Control1 DNA SARSCOV2	O22	-	-	ACTTCAGACTATTACCAGCTGTACTCAACT
Control2 DNA SARSCOV	O23	-	-	ACCGAAGTTTACTACCAGCTTGAGTCTACA
DNA4 MERS (Orf5)	O24	-	-	CCACTGTTTGACATGCGTTCTCACTTTATT
Control1 RNA SARSCOV	O25	-	-	CAUGGGAGAGAAAAAAAAUUUCUAAUUGUG
Control1 RNA MERS	O26	-	-	AUUUCAAGCGUUUGGUUUUUACCAAUUGCA
Control RNA	O27	-	-	UGCUAGAAAACCGCGUUUCUACGACUGGUG
Control2 RNA SARSCOV2	O28	-	-	ACUUCAGACUAUUACCAGCUGUACUCAACU
Control2 RNA SARSCOV	O29	-	-	ACCGAAGUUUACUACCAGCUUGAGUCUACA
RNA4 MERS (Orf5)	O30	-	-	CCACUGUUUGACAUGCGUUCUCACUUUAUU
MB WT (Spike)	O31	FAM	BHQ1	GTAACCAAGACCATTAGTGGGTTACGTCATCCGACGTCGGATGAC
MB B.1.1.7 (Spike)	O32	HEX	BHQ1	GTAACCAAGACCATAAGTGGGTTACGTCATCCGACGTCGGATGAC
RNA SC2 Wt	O33	-	-	CCAACCCACTAATGGTGTTGGTTAC
RNA SC2 Mut	O34	-	-	CCAACCCACTTATGGTGTTGGTTAC
Spike padlock RCA	O35	P	-	GTTCCAAGCATAAACAATTATCCCTATAGTGAGTCGTATTAGAATTTTC CCGGATCCTACATGCTGATTCTCTTCCT
Orfa8a padlock RCA	O36	P	-	CCTACTCTAATATACCATTATCCCTATAGTGAGTCGTATTAGAATTTTC CCAGTCGTACGCATGCTGATTTTCTAGCT
Universal RCA primer	O37	-	-	AATTCTAATACGACTCACTATAGG^G^
2019-nCoV_N1-F	O38	-	-	GACCCCAAAATCAGCGAAT
2019-nCoV_N1-R	O39	-	-	TCTGGTTACTGCCAGTTGAATCTG
2019-nCoV_N1-P-VIC	O40	VIC	3IABkFQ	ACCCCGCAT/ZEN/TACGTTTGGTGGACC

* = 2'-O-Methyl RNA modification

^ = 3' - Phosphorothioate modification

- Table S1. List of sequences used in this work

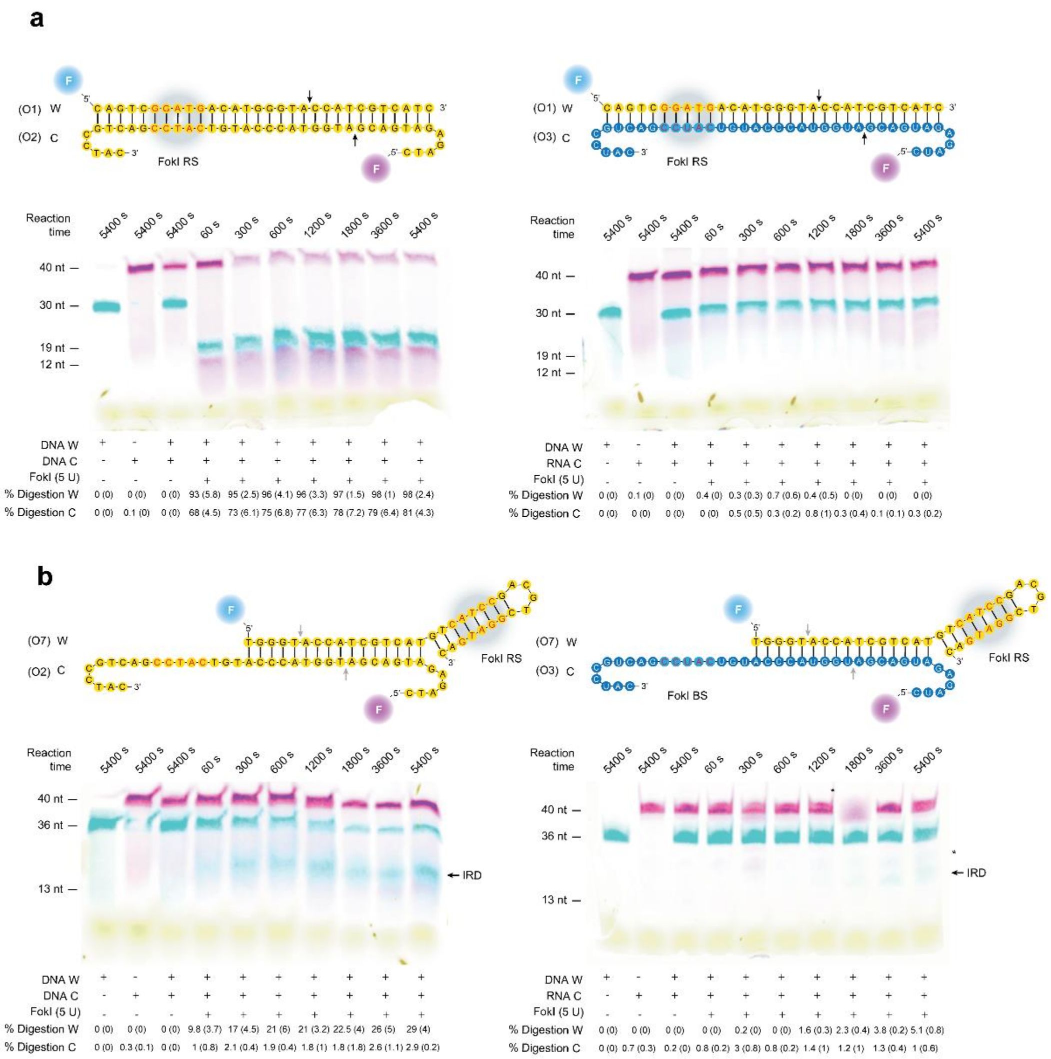

- Figure S1. FokI-assisted digestion of DNA duplexes and DNA:RNA hybrids (related to Figure 1). (a) Sequence of DNA duplexes (left panel) and DNA:RNA hybrids (right panel) indicating the 5' end labelling of the oligonucleotides with Cy5.5 (magenta) or IRD800 (blue). W and C represent the conventional positional nomenclature for the different molecules in the duplex or heteroduplex substrate (W = Watson strand, C = Crick strand), while yellow or blue dots denote the type of nucleic acid (DNA or RNA, respectively). FokI binding site is indicated by the shaded region, red nucleotides indicate the RE recognition site and arrows reflect the theoretical cleavage site in the different nucleic acid strands. Denaturing PAGE RNA gels show the restriction products obtained upon incubation of FokI and the corresponding substrate at different time points, measured in seconds. The left axis indicates the size (number of nucleotides) of the corresponding oligonucleotides or digested products. The legend shows the experimental combinations used in the restriction assay. Measurements indicate the average percentage of digestion and the standard deviation (in brackets) of the Watson or the Crick strand respectively. (b) Same as a, but the schema depicts the sequence and the structural hybridization properties of the hairpin oligonucleotide probes with a complementary DNA strand (left panel) or RNA strand (right panel). IRD indicates the products generated by FokI induced relaxation. For all panels, experimental conditions were performed in duplicate, and the quantification represents the average and the standard deviation of the detected bands as measured using the ImageStudio software.

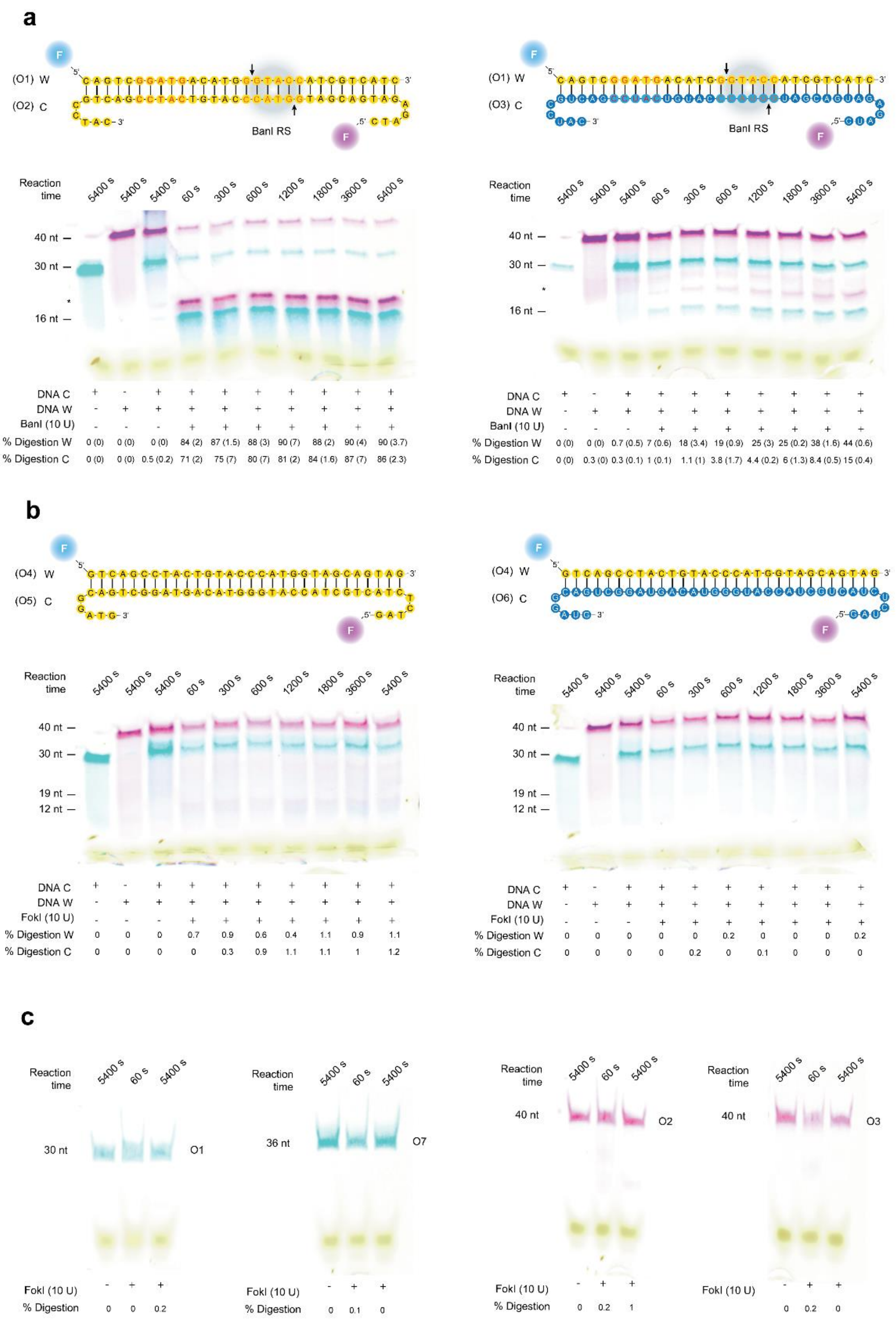

- Figure S2. Digestion of DNA duplexes and DNA:RNA hybrids mediated by BanI and FokI. (a) Sequence of DNA duplexes (left panel) and DNA:RNA hybrids (right panel) illustrating the 5' end labelling of the oligonucleotides with Cy5.5 (magenta) or IRD800 (blue) respectively. W and C indicate the conventional positional nomenclature for the different molecules in the duplex or heteroduplex substrate (W = Watson strand, C = Crick strand). Yellow or blue dots denote the type of nucleic acid (DNA or RNA, respectively). BanI binding sites are depicted by the shaded region and nucleotides in red indicate the exact sequence of the recognition site. Denaturing PAGE RNA gels show the restriction products obtained upon incubation of BanI and the corresponding substrate at different time points. The left axis indicates the size (number of nucleotides) of the

corresponding RE products. The legend reflects the experimental combinations used in the restriction assay. Measurements indicate the average percentage of digestion and the standard deviation (in brackets) of the Watson or the Crick strand respectively. The experiments from panel a were performed in duplicate for each of the conditions (b) Similar to a, but the schema depicts the sequence of the alternative DNA duplexes and DNA:RNA hybrids that lacked a FokI restriction site. Quantification represents the value of the bands detected as measured by the ImageStudio software. (c) Denaturing PAGE gels show the restriction products obtained upon incubation of FokI and each of the different individual substrates for 90 min at 37ºC. The size and the code of the corresponding DNA or RNA oligonucleotides is indicated. The legend shows the experimental combinations used in the restriction assay and measurements indicate the percentage digestion of the probes at the end of the reaction.

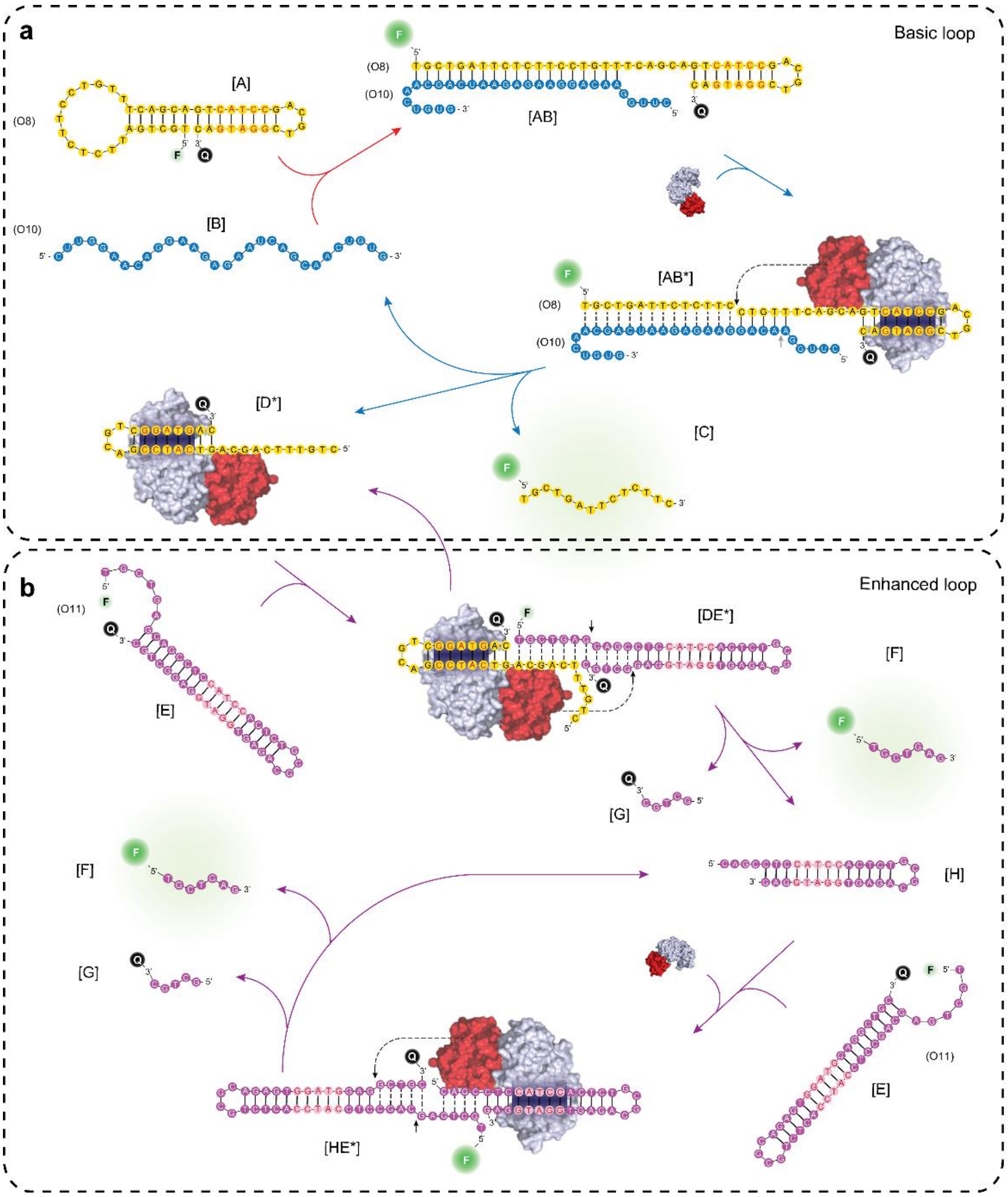

- Figure S3. Working principle of FokI-assisted digestion method. (a and b) Schema depicting the different stages of the signal amplification reaction. In the presence of a target molecule, the dumbbell-like oligonucleotide triggers the hybridization step. Depending on the number of components present in the reaction, a cascade of different feedback loops may be activated, resulting in the enhancement of the fluorescent signal of the assay. Red arrows represent the reaction obtained from a conventional hybridization assay. Blue arrows indicate the reaction steps generated in the basic FokI signal amplification assay. Magenta arrows represent the extended reaction steps achieved in the presence of a universal hairpin beacon oligonucleotide. Dashed circles indicate the molecular steps required for the basic (a) or enhanced (b) signal amplification reaction.

In a conventional hybridization assay, the absence of the target complementary molecule causes a Förster resonance energy transfer (FRET), which inhibits the fluorescence of the molecular beacon due to the proximity of the fluorescent dye-quencher pair. However, in the presence of the target sequence [B], one molecule of nucleic acid substrate can hybridize with one molecule of the dumbbell-like oligonucleotide (red arrow) generating a duplex/heteroduplex substrate [AB] and releasing the fluorescent

signal of the reporter molecule. The implementation of the FokI-assisted digestion system, by means of the combination of dumbbell-like structures and native FokI RE (blue arrow), may enhance the detection of nucleic acids as the cleavage reaction and the consequential release of the fluorescent signal is dependent on the hybridization between these oligonucleotides and the target sequence [AB*]. The asymmetric cleavage performed by FokI in this context might allow for the rapid release of the fluorescent portion of the probe [C] and the potential recirculation of the target sequence [B] for further rounds of digestion, thus improving on the 1:1 relationship observed with conventional hybridization assays.

To achieve greater enhancement of the signal amplification properties of the system, we also implemented an additional strategy for signal amplification mediated by a DNA machine, as previously postulated by Weizmann and coworkers[6]. In this context, the byproducts from the FokI-assisted reaction [D*] can still be used for further rounds of digestion in the presence of universal hairpin beacon oligonucleotides [E] (magenta arrow). These sequences are labelled with fluorescent dyes and quencher molecules at their 5' and 3' ends, respectively and contain an internal FokI recognition site and a 5' end overhang that can hybridize with the resulting product of the FokI-assisted reaction [D*]. The resulting substrate [DE*] can be further digested by circulating FokI RE units, releasing the fluorescent part of the universal hairpin beacon oligonucleotide [F] and generating two different byproducts [G and H]. As one of the byproducts [H] still retains a FokI recognition site and a 5' overhang which can hybridize with remaining universal hairpin beacon oligonucleotides [E], the new set of actionable substrates [HE*] can be further digested by FokI RE, generating the aforementioned G and H byproducts and creating a self-sustaining loop for signal amplification purposes.

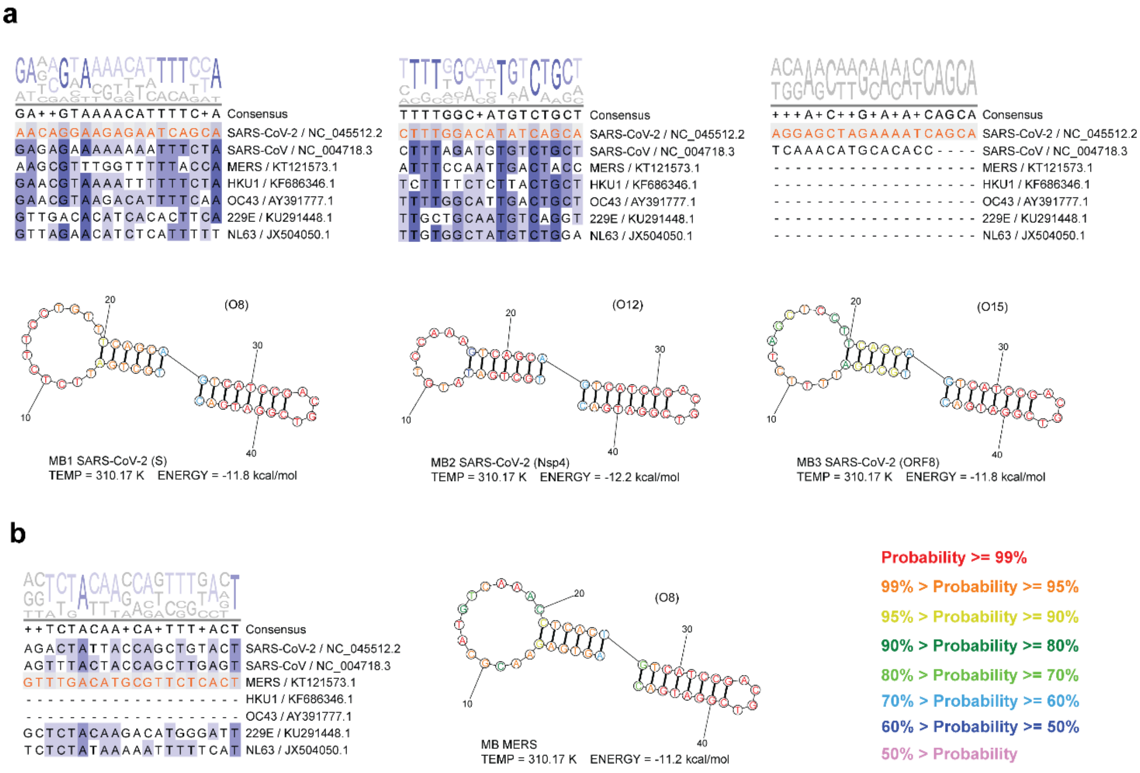

- Figure S4. Design of target DNA/RNA sequences and reporter oligonucleotides. (a) Upper panel represents the result of a Multiple Sequence Alignment (MSA) that includes the SARS-CoV-2 sequences targeted by the hairpin oligonucleotide probes (grey) and the sequences of 6 other human Beta coronaviruses. From left to right, MSAs corresponding to the Spike, Nsp4 and Orf8 regions, respectively. The lower panel depicts the sequence and the predicted structure of the dumbbell-like oligonucleotides as obtained from the RNAstructure server. The colour scale indicates the probability of the structure being present at a given nucleotide, as indicated in the bottom right legend. The lowest free energy for the different structures and the hybridization temperature are also indicated. (b) Similar to a, but the graph represents the MSA and the structure of the hairpin oligonucleotide probe targeting the spike region of MERS.

a

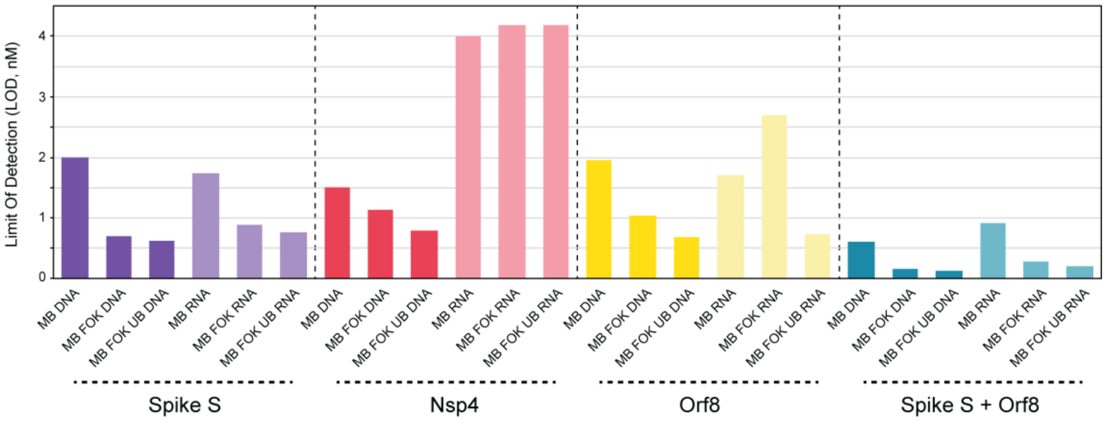

- Figure S5. LOD of the different reactions tested. (a) Bar plot represents the limit of detection (nM range) for the different conditions (DNA or RNA) and sequence combinations as calculated from the experimental data (see Methods section). MB @NA – Conventional hybridization approach. MB FOK @NA – Basic FokI-assisted signal amplification method. MB FOK UB @NA – Extended FokI-assisted signal amplification assay.

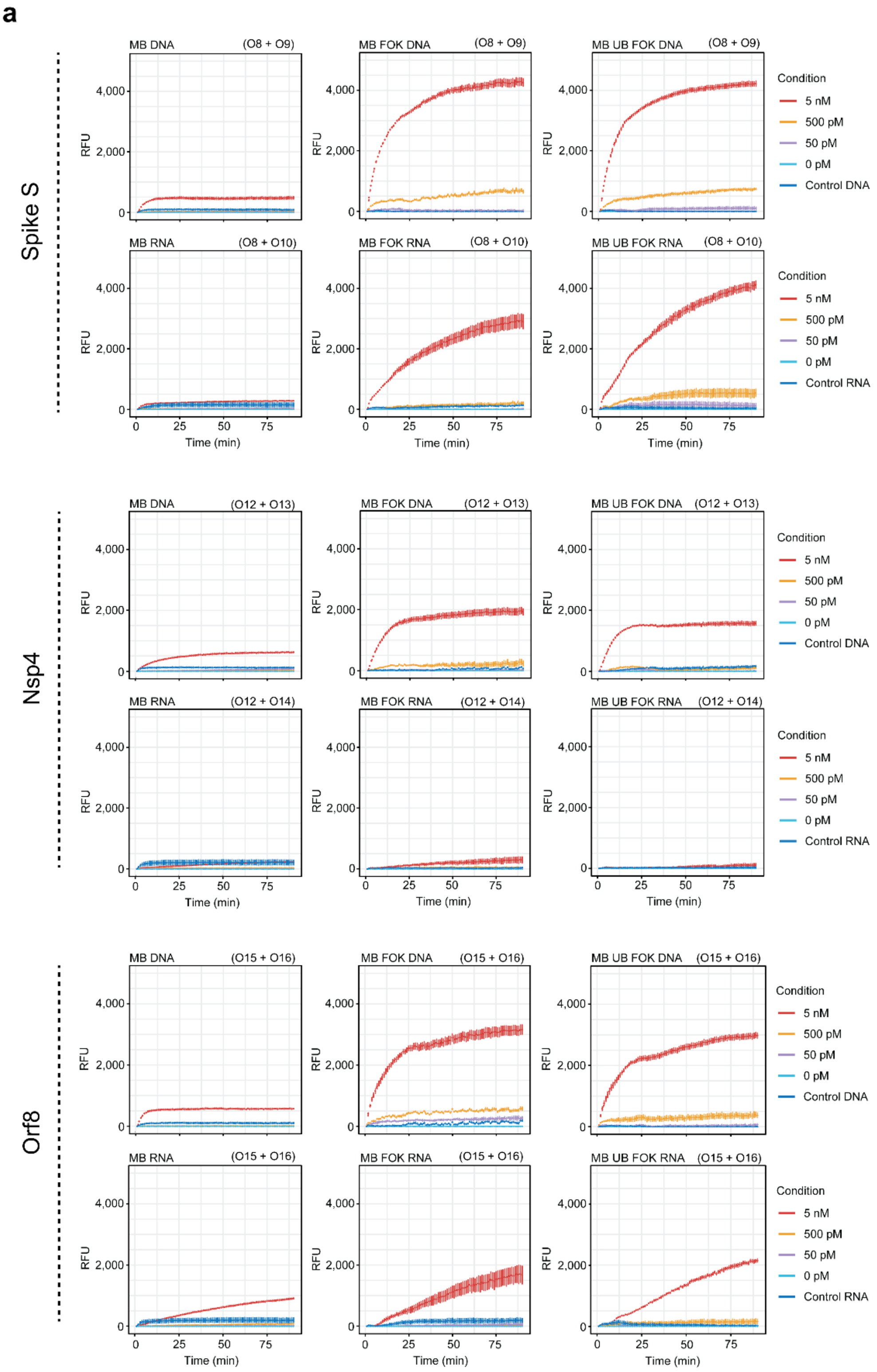

- Figure S6. Enhanced detection of individual SARS-CoV-2 sequences. (a) Line plots showing the real-time fluorescence detection measurements as indicated in Figure 3B, but for the case of the dumbbell-like oligonucleotides designed against Spike (upper graph), Nsp4 (middle graph) and Orf8 (lower graph) regions. Different concentrations of DNA (upper panels) or RNA (lower panels) were used, as previously indicated. For all the experiments, lines represent the averaged signal detection at a given time point and error bars indicate the standard deviation of 2 independent experiments performed in duplicate (n = 4).

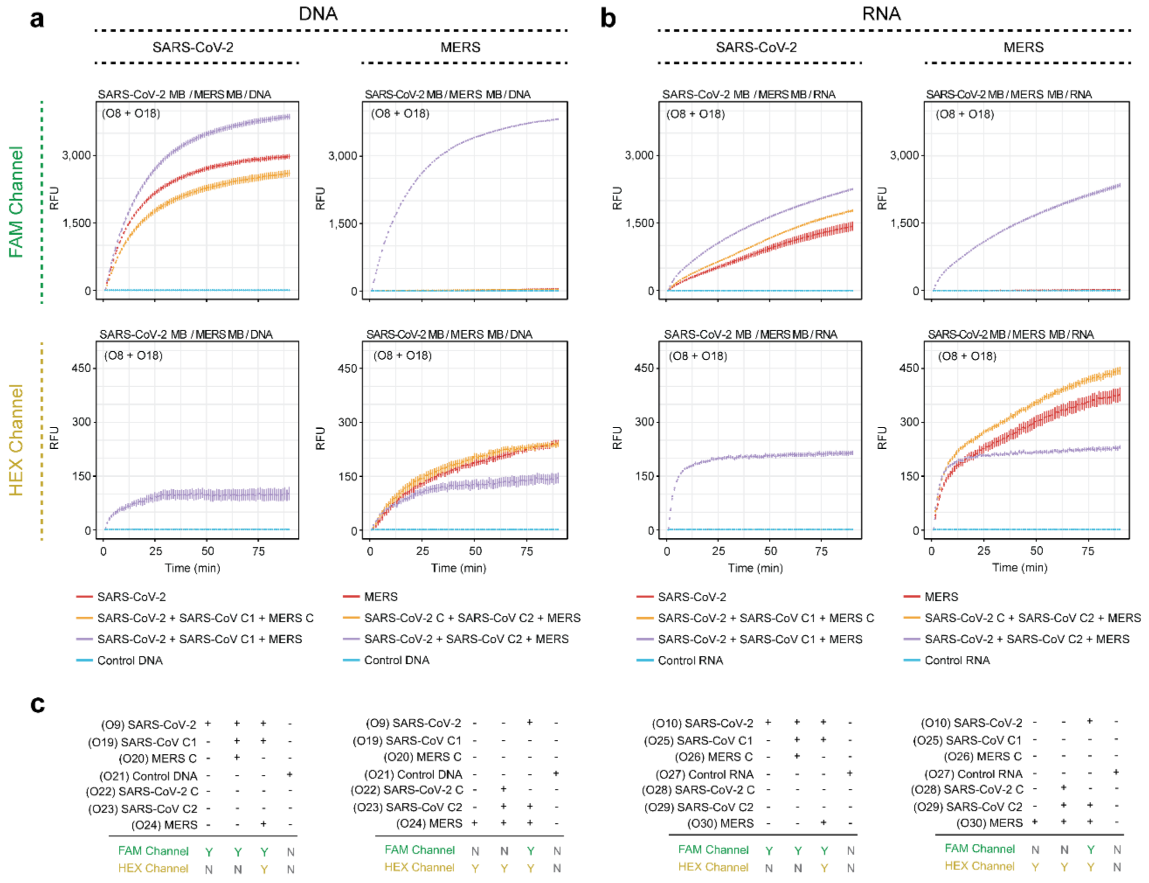

- Figure S7. FokI-assisted signal amplification allows the simultaneous detection of multiple targets. (a) Line plots indicating the real-time fluorescence detection measurements of the basic FokI-assisted signal amplification assay simultaneously recorded in the FAM and the HEX channels and in the presence of both dumbbell-like oligonucleotides. Experiments were performed at a constant concentration of 5 nM for each of the DNA substrates indicated in the legend, with the exception of the unrelated control sequence which was assayed at a concentration of 50 nM. Line colours corresponding to the different DNA substrate combinations are shown in the inner legend. For all the experiments, the concentration of the reporter dumbbell-like oligonucleotides was kept at a constant concentration of 100 nM. Lines show the averaged signal detection at a given time point and error bars indicate the standard deviation of 2 independent experiments performed in duplicate (n = 4). (b) Same as a, but for the use of RNA substrates. (c) For proper interpretation purposes, the schema summarizes the detection results obtained with the aforementioned combination of DNA or RNA substrates and the combination of reporter dumbbell-like oligonucleotides in each of the detection channels.

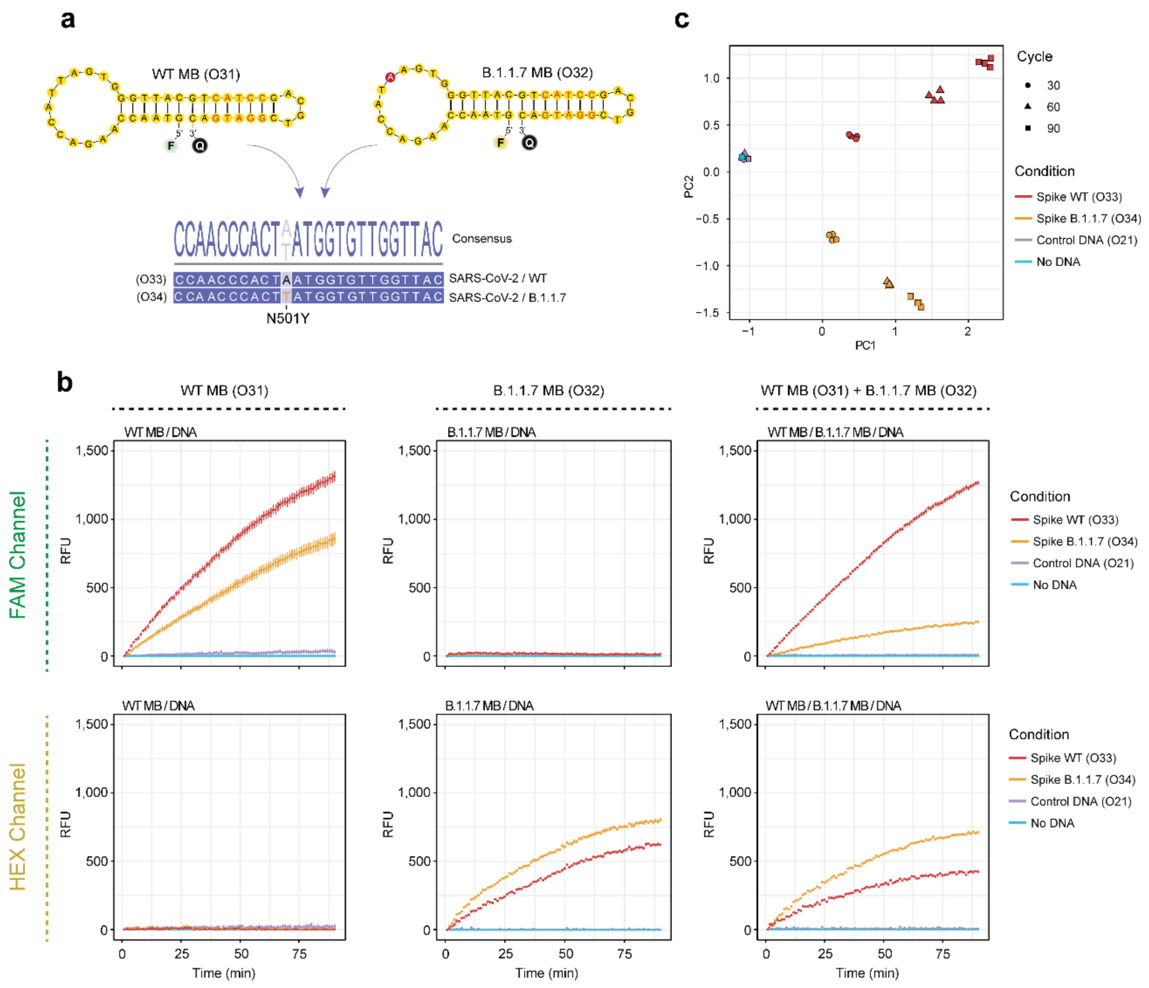

- Figure S8. FokI-assisted signal amplification discriminates between known SARS-CoV-2 variants. (a) Schema reflects the DNA sequence of the reporter dumbbell-like structures (green = 6-FAM, yellow = HEX, Q = BHQ1 quencher) and the sequence alignment of the wild type and the B.1.1.7 SARS-CoV-2 variants in the vicinity of the interrogated N501Y position. (b) Line plots indicating the real-time fluorescence detection measurements of the basic FokI-assisted signal amplification assay simultaneously recorded in the FAM and the HEX channels and in the presence of WT, B.1.1.7 or both reporter dumbbell-like oligonucleotides. Experiments were performed at a constant concentration of 5 nM for each of the DNA substrates indicated in the legend, with the exception of the unrelated control sequence which was assayed at a concentration of 50 nM. In these experiments, the concentration of each of the reporter dumbbell-like oligonucleotides was kept at a constant concentration of 100 nM. Lines represent the averaged relative fluorescence units detected at a given time point and error bars indicate the standard deviation of 2 independent experiments performed in duplicate (n = 4). (c) Principal component analysis of the aforementioned conditions in reactions containing both reporter dumbbell-like oligonucleotides reflecting differential signal trajectories for the different SARS-CoV-2 variants at the indicated time points. Combinations of dot colour and shape represent different substrates and incubation times, respectively.

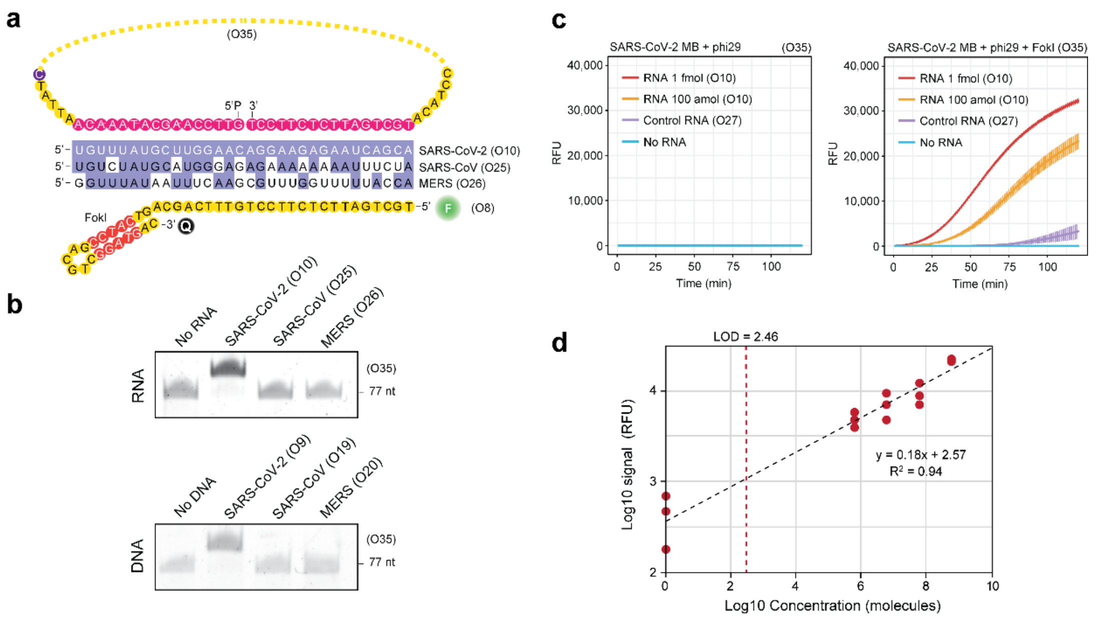

- Figure S9. Specificity and LOD of the RCA enhanced reaction. (a) Schema depicting the structure of a specific padlock probe (upper oligonucleotide) or dumbbell-like oligonucleotides (lower sequence) upon hybridization with its cognate target SARS-CoV-2 Spike RNA. Purple dots denote the hybridization sequence of the padlock probe with its RNA target and red dots highlight the binding site of the RE FokI. Middle sequences represents the results of a MSA against the Spike SARS-CoV-2 region. (b) Graphs illustrate the results of the ligation assay including padlock probes against the SARS-CoV-2 Spike region and their related complementary target RNAs (upper image) or DNAs (lower image), or oligonucleotides corresponding to orthologous regions of other SARS-like coronaviruses (SARS-CoV or MERS). The indicated oligonucleotides (250 nM each) were incubated with 12.5 units of SplintR ligase for 15 minutes at room temperature and ligation products were run on 12% denaturing PAGE gels for visualization purposes. The size of the linear padlock probe is indicated. (c) Line plots indicate the real-time fluorescence detection measurements of a RCA reaction coupled with the FokI-assisted signal amplification system in the presence of dumbbell-like oligonucleotides against the Spike region of SARS-CoV-2 in the absence (left panel), or in the presence (right panel) of 5 units of FokI RE. Reactions were monitored for 120 min and different amounts of target RNA substrates (1 fmol and 100 amol, 0 amol) or 1 fmol of an unrelated RNA sequence were used in the assay. Line colours corresponding to the different target concentrations are indicated in the inner legend. For all the experiments, the concentration of the reporter dumbbell-like oligonucleotide was kept at a constant concentration of 100 nM. Lines represent the averaged signal detection at a given time point and error bars indicate the standard deviation of 3 independent experiments. (d) Scatter plot depicting the linear relationship between increasing concentrations of RNA target (x-axis, Spike region, 1 amol, 10 amol, 100 amol, 1 fmol and unrelated control RNA), and the intensity of the fluorescent measurements obtained from the molecular coupling between RCA and the FokI-assisted signal amplification system (y-axis). Both relative fluorescence units and the number of target molecules included in the assay are represented in Log10 scale. Measurements were obtained after 60 min of reaction time. The theoretical limit of detection and other descriptive aspects of such linear relationship are also indicated.

Supplemental References

(1) Larkin, M. A.; Blackshields, G.; Brown, N. P.; Chenna, R.; McGettigan, P. A.; McWilliam, H.; Valentin, F.; Wallace, I. M.; Wilm, A.; Lopez, R.; Thompson, J. D.; Gibson, T. J.; Higgins, D. G. Clustal W and Clustal X Version 2.0. *Bioinformatics* 2007, *23* (21), 2947–2948.

(2) Waterhouse, A. M.; Procter, J. B.; Martin, D. M. A.; Clamp, M.; Barton, G. J. Jalview Version 2--a Multiple Sequence Alignment Editor and Analysis Workbench. *Bioinformatics* 2009, *25* (9), 1189–1191.

(3) Miller, J. R.; Delcher, A. L.; Koren, S.; Venter, E.; Walenz, B. P.; Brownley, A.; Johnson, J.; Li, K.; Mobarry, C.; Sutton, G. Aggressive Assembly of Pyrosequencing Reads with Mates. *Bioinformatics* 2008, *24* (24), 2818–2824.

(4) Reuter, J. S.; Mathews, D. H. RNAstructure: Software for RNA Secondary Structure Prediction and Analysis. *BMC Bioinformatics* 2010, *11*, 129.

(5) Leek, J. T.; Johnson, W. E.; Parker, H. S.; Jaffe, A. E.; Storey, J. D. The Sva Package for Removing Batch Effects and Other Unwanted Variation in High-Throughput Experiments. *Bioinformatics* 2012, *28* (6), 882–883.

(6) Weizmann, Y.; Cheglakov, Z.; Pavlov, V.; Willner, I. An Autonomous Fueled Machine That Replicates Catalytic Nucleic Acid Templates for the Amplified Optical Analysis of DNA. *Nat Protoc* 2006, *1* (2), 554–558.
